# Analysis of genomic rearrangements by using the Burrows-Wheeler transform of short-read data

**DOI:** 10.1186/1471-2105-16-S18-S5

**Published:** 2015-12-09

**Authors:** Kouichi Kimura, Asako Koike

**Affiliations:** 1 Biosystems Research Department, Center for Technology Innovation, Research & Development Group, Hitachi, Ltd., 1-280 Higashi-Koigakubo, Kokubunji, 185-8601 Tokyo, Japan

**Keywords:** sequence analysis, Burrows-Wheeler transform, genomic rearrangement, structural alteration, structural variation, breakpoint, cancer genome, short reads, next generation sequencing

## Abstract

**Background:**

The potential utility of the Burrows-Wheeler transform (BWT) of a large amount of short-read data ("reads") has not been fully studied. The BWT basically serves as a lossless dictionary of reads, unlike the heuristic and lossy reads-to-genome mapping results conventionally obtained in the first step of sequence analysis. Thus, it is naturally expected to lead to development of sensitive methods for analysis of short-read data. Recently, one of the most active areas of research in sequence analysis is sensitive detection of rare genomic rearrangements from whole-genome sequencing (WGS) data of heterogeneous cancer samples. The application the BWT of reads to the analysis of genomic rearrangements is addressed in this study.

**Results:**

A new method for sensitive detection of genomic rearrangements by using the BWT of reads in the following three steps is proposed: first, breakpoint regions, which contain breakpoints and are joined together by rearrangement, are predicted from the distribution of so-called discordant pairs by using a kind of the conjugate gradient method; second, reads partially matching the breakpoint regions are collected from the BWT of reads; and third, breakpoints are detected as branching points among the collected reads, and their precise positions are determined. The method was experimentally implemented, and its performance (i.e., sensitivity and specificity) was evaluated by using simulated data with known artificial rearrangements. It was applied to publicly available real biological WGS data of cancer patients, and the detection results were compared with published results.

**Conclusions:**

Serving as a lossless dictionary of reads, the BWT of short reads enables sensitive analysis of genomic rearrangements in heterogeneous cancer-genome samples when used in conjunction with breakpoint-region predictions based on a conjugate gradient method.

## Background

### Burrow-Wheeler transform

Suffix trees, suffix arrays, and their variants have long been studied, over the past quarter of a century, in relation to diverse text-search problems. In particular, the Burrows-Wheeler transform (BWT) [1], aka FM index [2], is one of the most memory-efficient variations with rich order-preserving (a kind of homomorphic) properties in various situations.

Recently, the advent of so-called next-generation sequencers (NGS) has posed a challenging problem for developing ultrafast genome-mapping tools that can cope with the unprecedented flood of short-read data [3]. Today, the problem has already been almost solved satisfactorily by popular short-read mapping tools, many of which owe their superior performance to the BWT of reference genomes [4,5]. In other words, the great usefulness of the BWT of reference genomes has been undoubtedly demonstrated.

In contrast, except for some pioneering works, the utility of the BWT of a large amount of short-read data remains largely unexplored. Efficient methods for computing the BWT of a large amount of short-read data, typically larger than 100 Gbp (giga base pairs), were presented in [6,7]. Simpson *et al*. [8] proposed an efficient de novo assembler using the compressed BWT of reads. Cox *et al*. [9] proposed large-scale compression (using the BWT of reads) of genomic sequence data. Janin *et al*. [10] proposed adaptive large-scale reference-free compression (also using the BWT of reads) of base-calling quality scores. In our previous studies, the authors showed that the BWT of reads enables ultrafast analysis of single nucleotide polymorphisms (SNPs) [7].

The BWT of short-read data basically serves as a lossless dictionary of the read data, unlike the heuristic and lossy reads-to-genome mapping results conventionally obtained in the first step of sequence analysis. Thus, it is naturally expected to lead to development of sensitive analysis methods, as will be demonstrated in this study.

### Genomic rearrangements and breakpoints

Genomic rearrangements are variations of genomic sequence on a large scale, typically 1 Kbp (kilo base pairs) or more, as opposed to single nucleotide variations (SNV) and small indels (insertions or deletions). Inherited and acquired rearrangements are referred to as "structural variations" and "structural alterations", respectively. Structural alterations are often found in many cancers and have a great influence on various disease states.

In cancer studies, matched tumor and normal samples are comparatively analyzed because somatic (acquired) structural alterations are only found in the cancer samples, while germline (inherited) structural variations are similarly found in both samples. However, tumor samples are usually heterogeneous due to instability of genomic DNA, and partially contain normal cells. Therefore, their analysis requires high sensitivity. Furthermore, recent studies have revealed that some cancer genomes harbor very complicated rearrangements resulting from discontinuous steps of carcinogenesis processes known as chromothripsis [11] and chromoplexy [12,13].

Genomic rearrangements basically occur as follows: genomic DNA sequences are split into fragments at certain *breakpoints*, and the fragments are re-joined across the breakpoints in different orders and in different orientations from those before the splitting. These events occur on a large scale, typically larger than 1 Kbp, namely, much longer than 100 bp, a typical length of short reads.

### Analysis of breakpoints from short-read data

Breakpoints associated with large-scale genomic rearrangements can be indirectly detected from paired-end short-read whole-genome sequencing (WGS) data. Paired-end reads are obtained by sequencing both ends of a genomic DNA fragment, customarily called *an insert*. The inserts are typically obtained by sonication of a DNA sample, and they have approximately the same length, which is usually somewhat larger than twice the read length, but much smaller than the scale of the genomic rearrangement.

When an insert does not contain any breakpoints, the paired-end reads satisfy the *paired-end mapping condition*, namely, they are mapped onto the reference genome in an inward orientation and apart from each other by a distance equal to the insert length. Such a pair of reads is referred to as *an accordant pair*. On the other hand, when an insert contains a breakpoint at which distant parts of the genome are concatenated, the paired-end reads are mapped to the corresponding parts, and the paired-end mapping condition is violated. Such a pair of reads is referred to as *a discordant pair*.

Discordant pairs are easily found from short-read data by using standard paired-end mapping tools such as BWA [4]. Subsequently, the existence of breakpoints and their approximate positions can be predicted from the discordant pairs [14-17]. However, their precise positions, at single-base-level resolution, cannot be determined.

Split reads are reads that contains breakpoints. They are so called because they can be split into separate parts that are mapped onto different parts of the genome. The precise positions of breakpoints are immediately given by the positions of the splits. It is possible to find split reads by using standard mapping tools -- for example, collecting reads that can be mapped onto the genome only partially with significant unmapped overhangs, and mapping the overhangs onto the genome. However, the overhangs are often too short to be mapped unambiguously. Moreover, the number of split reads is much smaller than the number of discordant pairs because these numbers are roughly proportional to read length and insert length. Therefore, it is difficult to sensitively detect breakpoints on the basis of split reads.

Recent tools for analyzing genomic rearrangement can sensitively detect breakpoints and precisely determine the positions on the basis of integrated analysis of discordant pairs, split reads, and others [15-17]. In this study, a method for sensitively detecting break-points and precisely determining their positions, by using the discordant pairs and the dictionary of reads but without using the split reads, is proposed.

## Methods

### Conceptual method for confirming known breakpoints

Confirming the existence of known breakpoints by using a dictionary of reads is conceptually simple and straightforward on the assumption that the exact breakpoint positions are precisely known. Namely, it is only necessary to confirm the existence of read fragments going through the breakpoints. This confirmation can be done immediately by using a query given by a concatenation of genomic subsequences that are to be concatenated by rearrangement.

More specifically, let *G *= ... *a|b *... *c|d *... be a genomic sequence with a pair of known breakpoints (indicated by "*|*"), where *a, b, c *and *d *denote subsequences adjacent to the breakpoints, and "..."s denote the remaining sequences. For simplicity, assume that there are no other breakpoints. The known breakpoints can relate to four different rearrangements corresponding to four different concatenations, namely, *ad, ac', cb*, and *d'b*, or their reverse complements, *d'a', ca', b'c'*, and *b'd*, where *a' *denotes the reverse complement of *a *and so on. Existence of these concatenations can be checked immediately by using exact matching with backward search [4] (essentially, by using so-called LF functions [2] or rank functions [18]). The chosen length of the adjacent subsequence should be large enough so as to avoid ambiguous false detection.

In practice, however, most of known breakpoints are not given in terms of exact base positions but in terms of approximate positions relative to nearby genes or exons. In fact, even if similar disease-related fused genes are found in patients with the same cancer subtype, the precise breakpoint positions differ from patient to patient [13]. It is therefore necessary to make a number of queries according to a varying combination of possible precise breakpoint positions. Specifically, *ad *differs according to the chosen ends of *a *and chosen heads of *d*. Moreover, as is often the case, if an unknown short sequence is intervened between the breakpoints in the rearrangement, for example, *asd *instead of *ad *for some unknown short sequence *s*, it is necessary to make a number of queries, *asd*, according to varieties of the intervening *s*. Thus, it is not practical to make a large number of queries directly corresponding to all possible cases.

### Overview of the proposed method in practical use

To exploit the dictionary of reads in practice, it is important to make a limited number of effective queries that are likely to extract useful information from the dictionary. In particular, effective queries should be chosen from genomic regions where breakpoints are likely to exist. Additionally, breakpoint-containing queries should be avoided because they have a combinatorially increasing and unaffordable number of variations. Accordingly, the proposed method is composed of the following three steps.

1 Predict *breakpoint regions *that are likely to contain breakpoints and be joined by rearrangement.

2 Scan the breakpoint regions with a sliding window from right to left, collect all the read fragments exactly matching the window, and subsequently collect all possible leftward extensions of the fragments.

3 Identify breakpoints as branching points among the extensions.

The dictionary of reads is effectively used in the second step. The most time-consuming steps are the last two steps; therefore, it is important to reduce the size of the breakpoint regions as much as possible. The first step further consists of the following three sub-steps:

1a Collect discordant pairs by using a standard paired-end mapping tool.

1b Get rough breakpoint regions by means of clustering the discordant pairs.

1c Narrow down the breakpoint region in each cluster on the basis of a detailed analysis of the distribution of discordant pairs.

The first two sub-steps are basically similar to known methods in previous studies [14-17], while the third sub-step is first introduced in this study. These sub-steps and the following main steps are described in the following subsections.

### Collection of discordant pairs

The paired-end short reads are mapped onto a reference genome by using a standard paired-end mapping tool, BWA [4]. Discordant pairs are extracted from the results in the SAM (sequence alignment/map) format [19] by reference to bitwise flags indicating whether paired-end mappings are accordant or discordant. Only unambiguous mapping results with Phred-scaled mapping-quality score not less than 30 are employed.

### Two-dimensional clustering of discordant pairs

A global coordinate system is defined on the whole reference genome sequence. Namely, DNA sequences from all chromosomes are concatenated into a single DNA sequence, *G*, with a punctuation symbol "$" in between them; and each base position in each chromosome is specified by a global coordinate indicating the position in the concatenated sequence. Thus, all chromosomal positions are mutually comparable according to the global coordinates.

Each discordant pair of reads is represented by a point, (*x, y*), in a two-dimensional region, *G×G*, where *x *and *y *are the global coordinates of the mapped positions of the first bases of the reads, and, for disambiguation, *x < y *is assumed because *x *and *y *can be swapped otherwise. Thus, discordant pairs are identified with the corresponding points in *G × G*.

The discordant pairs are scattered very sparsely as a whole in *G × G *because it is vast, and those associated with the same breakpoint are clustered together within a distance of the insert length. Therefore, it is easy and straightforward to extract such clusters.

As an example of extraction procedure, sort the points (discordant pairs) according to the first coordinates, and classify them into groups so that the differences between the first coordinates in the same (different) groups are less (larger) than the insert length; subsequently, sort the points in each group according to the second coordinates, and further classify them into subgroups so that the differences between the second coordinates in the same (different) subgroups are less (larger) than the insert length; repeat such classification several times, and obtain the desired clusters.

As for matched tumor and normal samples, clusters of discordant pairs associated with somatic breakpoints, simply referred to as *somatic clusters *here-after, exclusively consist of those from the tumor sample, while clusters of discordant pairs associated with germline breakpoints, simply referred to as *germline clusters*, are mixtures of those from both tumor and normal samples. Therefore, clusters are judged to be somatic if the rate of discordant pairs from the tumor is high, e.g., 90% or more, and judged to be germline otherwise.

### Prediction of breakpoint regions

Breakpoints are also represented by points in *G × G *in a natural way. Namely, when *x*_0 _and *y*_0 _are breakpoints originally apart from each other on *G *and are joined together by rearrangement, the pair of breakpoints is represented by point (*x*_0_*, y*_0_), where, for disambiguation, *x*_0 _*< y*_0 _is again assumed. Thus, breakpoints are identified with the corresponding points in *G × G*.

When discordant pairs associated with the same breakpoint are clustered together, the breakpoint is within the insert length in *G × G *from the cluster. Therefore, the cluster of discordant pairs roughly indicates the breakpoint region. Furthermore, the region can be reduced drastically on the basis of detailed analysis of the relationship between the distribution of breakpoints and that of discordant pairs.

#### Forward relationship -- from breakpoints to discordant pairs

**Figure 1:**
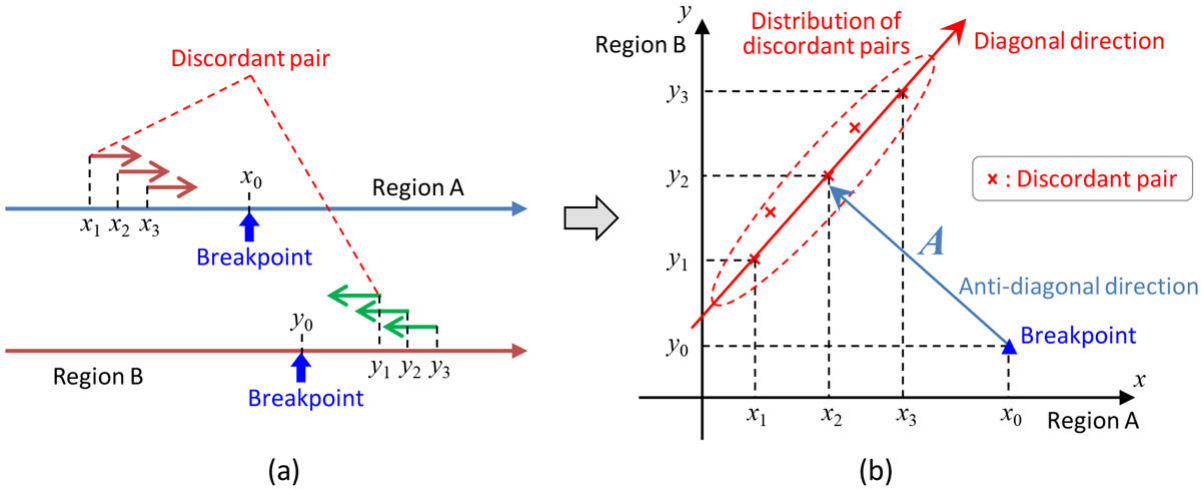
**Distribution of discordant pairs associated with a breakpoint**. For each breakpoint, associated discordant pairs are distributed in a belt extended in the diagonal direction (at an angle of 45 degrees) located in the anti-diagonal direction (at an angle of 135 degrees) from the breakpoint. This corresponds to one of the four different conditions on the ordering of coordinates; namely, *x_i _< x*_0 _*< y*_0 _*< y_i _*for 1 *≤ i ≤ p*. As for three remaining conditions, relative positions of the associated discordant pairs are rotated 90, 180, or 270 degrees around the breakpoint.

Assume that discordant pairs (*x*_1_, *y*_1_), (*x*_2_, *y*_2_), ..., (*x_p_, y_p_*) are associated with breakpoint (*x*_0_, *y*_0_). As explained earlier, in disregard of possible short intervening sequences, there are four essentially different rearrangements associated with breakpoint (*x*_0_, *y*_0_). They correspond to four different conditions on the ordering of coordinates: *x_i _< x*_0 _*< y*_0 _*< y_i_, x*_0 _*< x_i _< y*_0 _*< y_i_, x_i _< x*_0 _*< y_i _< y*_0_, and *x*_0 _*< x_i _< y_i _< y*_0 _for 1 *≤ i ≤ p*. Here, only the first case is considered, since other cases can be treated similarly. Then, the discordant pairs are distributed in a belt in *G×G *extended in the diagonal direction and located in the anti-diagonal direction from the breakpoint (Figure 1).

In particular, when the inserts are obtained by sonication of a DNA sample, the distribution of the insert length is well-approximated by a Gaussian distribution, *N *(*L, σ*), where *L *and *σ *denote the mean and standard deviation. Then, discordant pair (*x, y*) associated with given breakpoint (*x*_0_, *y*_0_) is distributed such that

1 ℓ = (*x*_0 _- *x*) + (*y *- *y*_0_) is distributed according to *N *(*L, σ*).

2 *x*_0 _- *x *is uniformly distributed in interval (0, ℓ) on the condition that (*x*_0 _*− x*) + (*y − y*_0_) = ℓ.

The first condition refers to the distribution of insert length, and the second condition reflects an ideal condition that there is no sonication bias around the breakpoint.

Distribution of breakpoints is represented by vector ***x ***= (*x_ij_*) indexed by *i, j *∈ *G *with *i < j*, where *x_ij _*= 1 if a breakpoint exists at (*i, j*) ∈ *G × G*, and *x_ij _*= 0 otherwise. Then, distribution of the discordant pairs associated with these breakpoints is given by ***y *= *Ax***, where ***A ***is a stochastic matrix determined by the distribution of the insert length. More precisely, given a discordant pair, the probability that it is found at (*i, j*) ∈ *G × G *is given by *y_ij_*/*n*, a component of *y *= (*y_ij_*) divided by the number of breakpoints, *n*.

***A ***represents a linear transformation of a distribution of breakpoints into a distribution of discordant pairs. In particular, when the insert length is distributed according to a Gaussian distribution, it is given by a composition of three basic linear transformations:

(1) $$A=G_{\sigma }U_{L}T_{L}$$

where ***G_σ _***represents a two-dimensional Gaussian diffusion with standard deviation *σ*, ***U_L _***represents a one-dimensional uniform diffusion in finite length $\pm L/\sqrt{2}$ in the diagonal direction, and ***T_L _***represents a parallel translation in the upper-left anti-diagonal direction by distance $L/\sqrt{2}$. These basic linear transformations are mutually commutative. In practice, these linear transformations are discretized so that they work on grid points in *G × G*.

#### Backward relationship -- from discordant pairs to breakpoints

An empirical distribution of discordant pairs found by analysis of short-read data is represented by vector *y *= (*y_ij_*) indexed by *i, j *∈ *G *with *i < j*, where *y_ij _*is the number of discordant pairs found at (*i, j*) ∈ *G × G*. Note that it is not normalized as a probability distribution. Since only a limited number of discordant pairs can be obtained from a finite amount of short-read data, the empirical distribution varies from sequencing to sequencing even for the same sample, and approximates to the unknown true distribution.

In principle, the distribution of breakpoints can be obtained by solving the following equation:

(2) Ax=cy

where *c *is a normalizing constant. However, the equation can be solved more easily by a kind of conjugate gradient method. Namely, by multiplying both sides of the equation by ***A***^∗^, the conjugate linear transformation (transposed matrix) of ***A***, from the left,

(3) $$A^{*}Ax=cA^{*}y$$

is obtained. The obtained equation is conventionally referred to as *normal equation*. It is noteworthy that the composite transformation on the leftside is positive semi-definite and self-adjoint: $x\cdot A^{*}Ax=||Ax|{|}^{2}\geq 0$ for any ***x ***and (***A***^∗^***A***)^∗^ = ***A***^∗^***A***. Intuitively, ***A***^∗^***A ***is a "blurring" transformation.

In particular, when the insert length is distributed according to a Gaussian distribution, the composite transformation is given by

(4) $$A^{*}A={\left(G_{\sigma }U_{L}T_{L}\right)}^{*}G_{\sigma }U_{L}T_{L}=G_{2\sigma }U_{L}^{2}.$$

This is a two-dimensional diffusion symmetric both in the diagonal and anti-diagonal directions.

#### Prediction of breakpoint regions by using the conjugate

The breakpoint regions, where *x_ij _*is significantly large, are blurred by ***A***^∗^***A ***and expanded to wider regions in which *z_ij_*, where *z_ij _*is a component of ***z ***= ***A***^∗^***y***, is significantly large. Therefore, a prediction of breakpoint regions is given by regions where *z_ij _*is significantly large. Since the blurrings occur locally, ***z ***= ***A***^∗^***y ***in each cluster can be computed independently.

**Figure 2:**
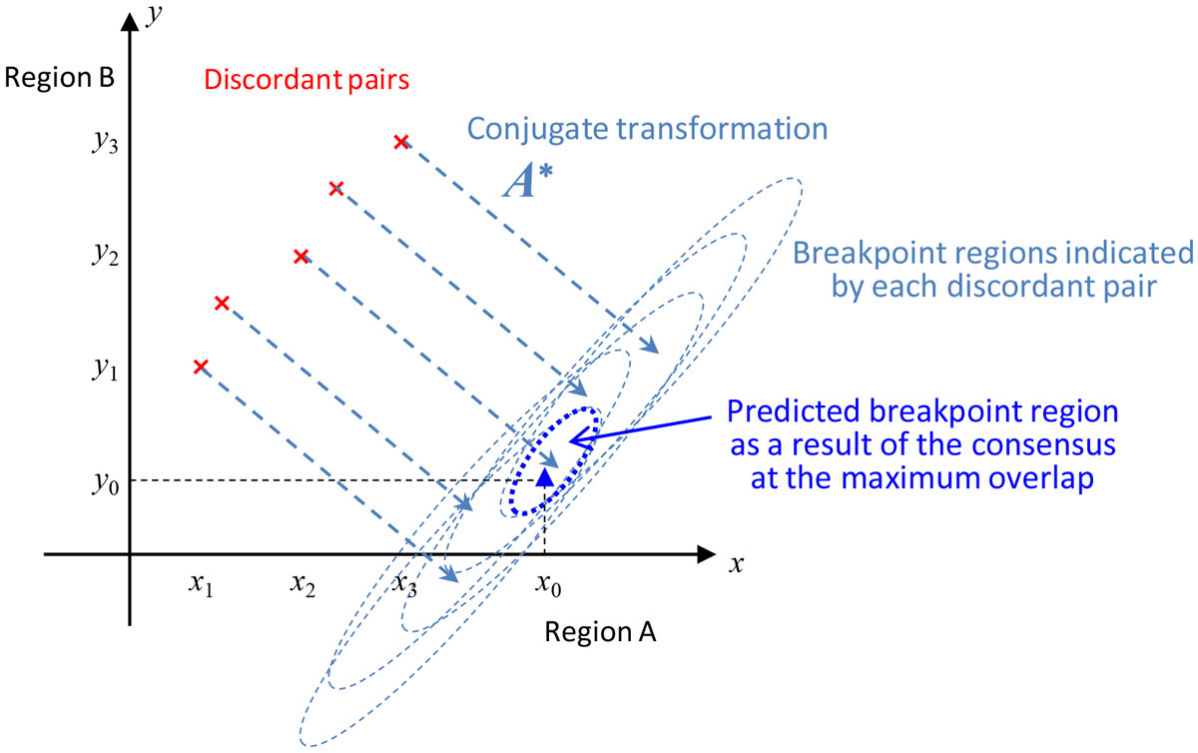
**Conjugate transformation of an empirical distribution of discordant pairs**. When discordant pairs are associated with an unknown breakpoint, each discordant pair indicates, through the conjugate transformation, a region where the breakpoint likely to exist. A prediction of breakpoint region is therefore given by a result of consensus among them at the maximum overlap.

Incidentally, ***A***^∗^***y ***can be interpreted intuitively as follows. If there is only a single discordant pair associated with an unknown breakpoint, ***A***^∗^***y ***indicates a region where the breakpoint is likely to occur (Figure 2); more precisely, *z_ij_*, a component of *z *= ***A***^∗^***y***, is the likelihood that the breakpoint occurs at position (*i, j*) ∈ *G × G*. Thus, the breakpoint is most likely to occur at the maximum component of ***A***^∗^***y***. If there are multiple discordant pairs associated with the same unknown breakpoint, the breakpoint is most likely to occur in the overlap among the regions indicated by the discordant pairs (Figure 2).

### Collecting relevant read fragments and their extensions

To find breakpoints in the breakpoint regions, relevant read fragments that partially match with the regions are collected. If the matching length is too small, irrelevant read fragments are also collected with the desired ones. Therefore, the matching length is chosen to be somewhat (e.g., by three bases) larger than MLU (minimum length for uniqueness). MLU is defined on the reference genome as the minimum length of the subsequence starting from a given position and extending in a given direction such that the subsequence appears only once in both strands of the genome [7]. MLUs over the whole reference genome are efficiently computed, and the results are compactly represented [7].

**Figure 3:**
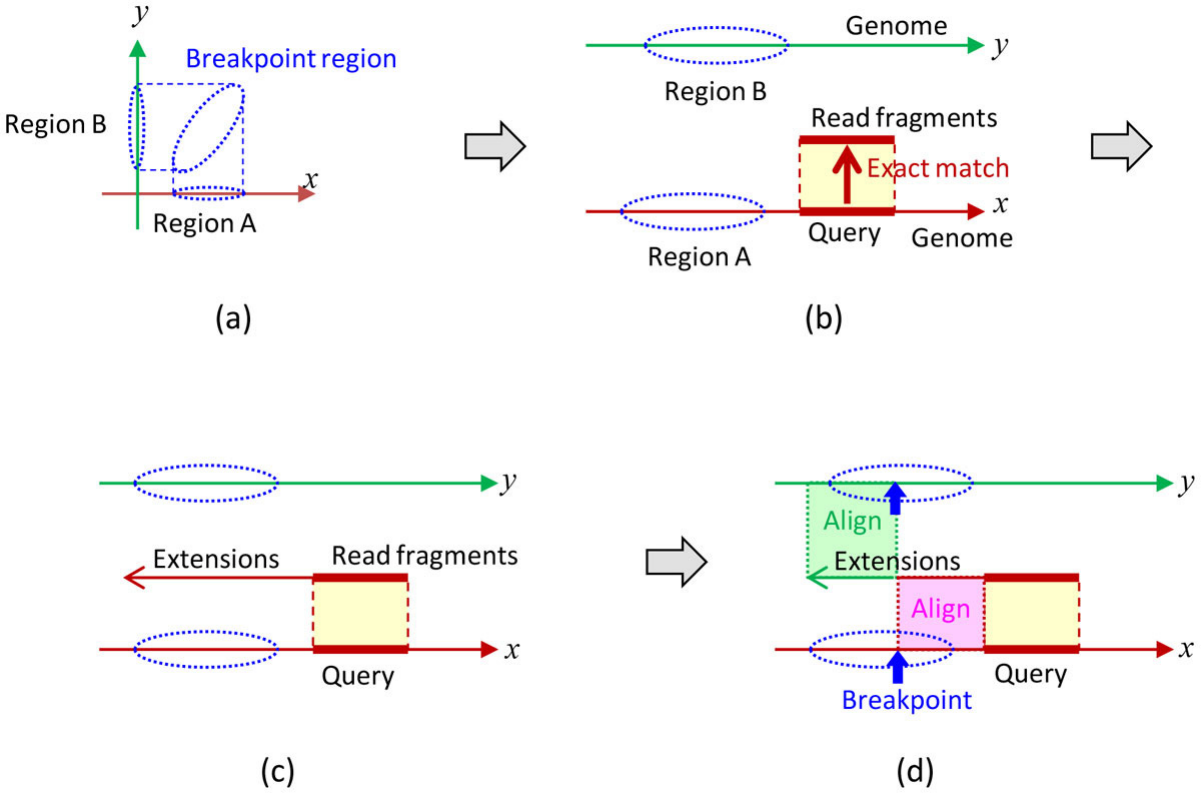
**Collecting partially matching read fragments and their extensions**. (a) regions A and B (projections of a two-dimensional breakpoint region along each dimension); (b) read fragments exactly matching a query in region A; (c) leftward extensions from the read fragments; and (d) comparison of an extension with each of regions A and B.

Figure 3 illustrates how the relevant read fragments and their subsequent extensions are collected. Regions A and B are the projections of a two-dimensional breakpoint region onto each dimension (Figure 3(a)).

The reference genome sequence around one of the projected regions, say, region A, is scanned from right to left, and a query is taken as a genomic subsequence of length MLU+(*x*) + *α *with the left end at *x*, where MLU^+ ^(*x*) denotes MLU at *x *in the positive (right-ward) direction, and *α *is a small positive constant (Figure 3(b)). All of the read fragments exactly matching the query are collected from the dictionary of reads by exact matching with backward search [1].

Similarly, all of the possible leftward extensions of the collected read fragments are obtained by exact matching with backward search (by recursive examinations of all four possible extensions with A, C, G, and T at each extended base position) (Figure 3(c)). The extensions are performed until the length of extension reaches a fixed value, e.g., 20.

Generally, the extensions are diverse as a result of SNPs, breakpoints, sequencing errors, and other events. In particular, when some of the extensions contain breakpoints joining regions A and B, they can be detected by aligning them with the reference genome sequences in each of regions A and B (Figure 3(d)).

### Detection of breakpoints from the extensions

**Figure 4:**
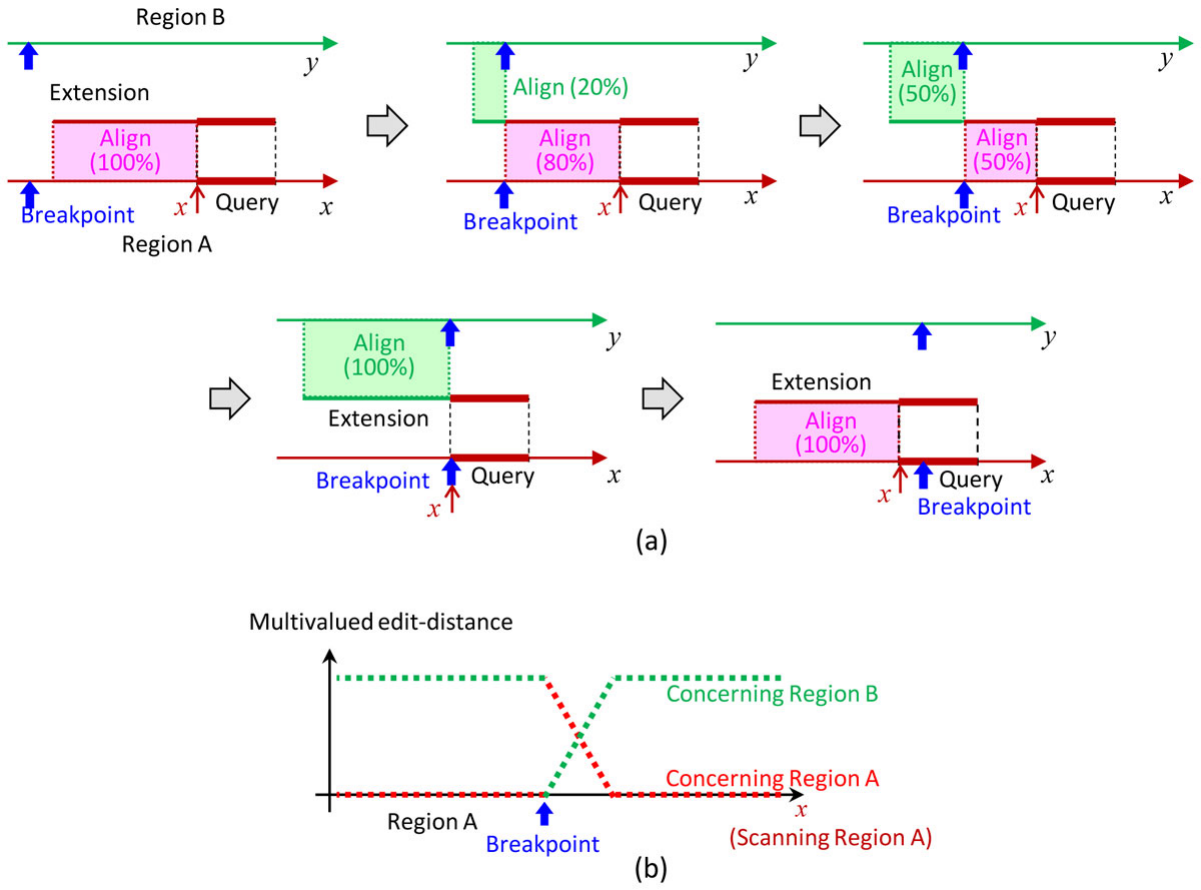
**Detection of a breakpoint in a breakpoint region**. (a) Optimal alignment of an extension with region A (B) changes while query position, *x*, scans region A from right to left. (b) Minimum edit distances given by the optimal alignment are plotted against scanning position *x*; a precise breakpoint position is indicated.

The extensions can be aligned efficiently with the regions A and B by a very fast dynamic programming algorithm based on bit-level parallelism [20]. Since the minimum edit distance attained at the optimal local alignment can be computed much faster than the optimal local alignment, which requires backtracking computations, it is advantageous to detect breakpoints only from the minimum edit distances. Figure 4 intuitively illustrates this detection.

While the left end of the query, *x*, scans from right to left a neighborhood of region A, the minimum edit distances between extensions (from the query in region A) and region A (B) are computed. In the simplest case where there is only a unique extension with a single breakpoint, they are given by the length of unaligned part of the extension in the optimal alignment (Figure 4(a)). Specifically, when at most *d *% of the extension can be aligned somewhere in region A (B), they are given by (1 *− d/*100)*e*, where *e *is the extension length. They are functions of *x *(Figure 4(b)), more precisely, multivalued functions of *x *because of the multiplicity of extensions. They are thus hereafter referred to as *a multivalued edit-distance function *concerning region A and that concerning region B.

When *x*, the left end of the query in region A, passes though a breakpoint, the edit-distance function changes drastically as shown in Figure 4(b). On the other hand, when breakpoints do not exist, such a drastic change never happens -- the multivalued editdistance function concerning region A remains zero, and that concerning region B remains large. Thus, breakpoints are detected from the multivalued editdistance function, and their precise positions are also obtained. To provide more confidence in the detection, similar analysis is performed once again with regions A and B swapped.

As for the analysis of matched tumor and normal samples, breakpoints are somatic if such a drastic change is found only in the tumor sample but not in the normal sample.

### Computation of the BWT of short-read data

The BWT of a large amount of short-read data, typically larger than 100 Gbp, is efficiently calculated by the BWT/WT algorithm [7], which basically follows the BCRext algorithm [6] with DNA sequences represented by wavelet trees [21].

## Results and discussion

### Examples of analysis of real biological data

The proposed method was experimentally implemented for proof of concept. To demonstrate how the method actually works for real biological data, several examples of analysis are presented here. The data are publicly available WGS data of matched tumor and normal samples, SRR559219 and SRR550170 in the NCBI Sequence Read Archive (SRA). The samples were taken from excised tumor tissue and blood from the same patient with gastric cancer [22].

#### Two-dimensional clusters of discordant pairs

**Figure 5:**
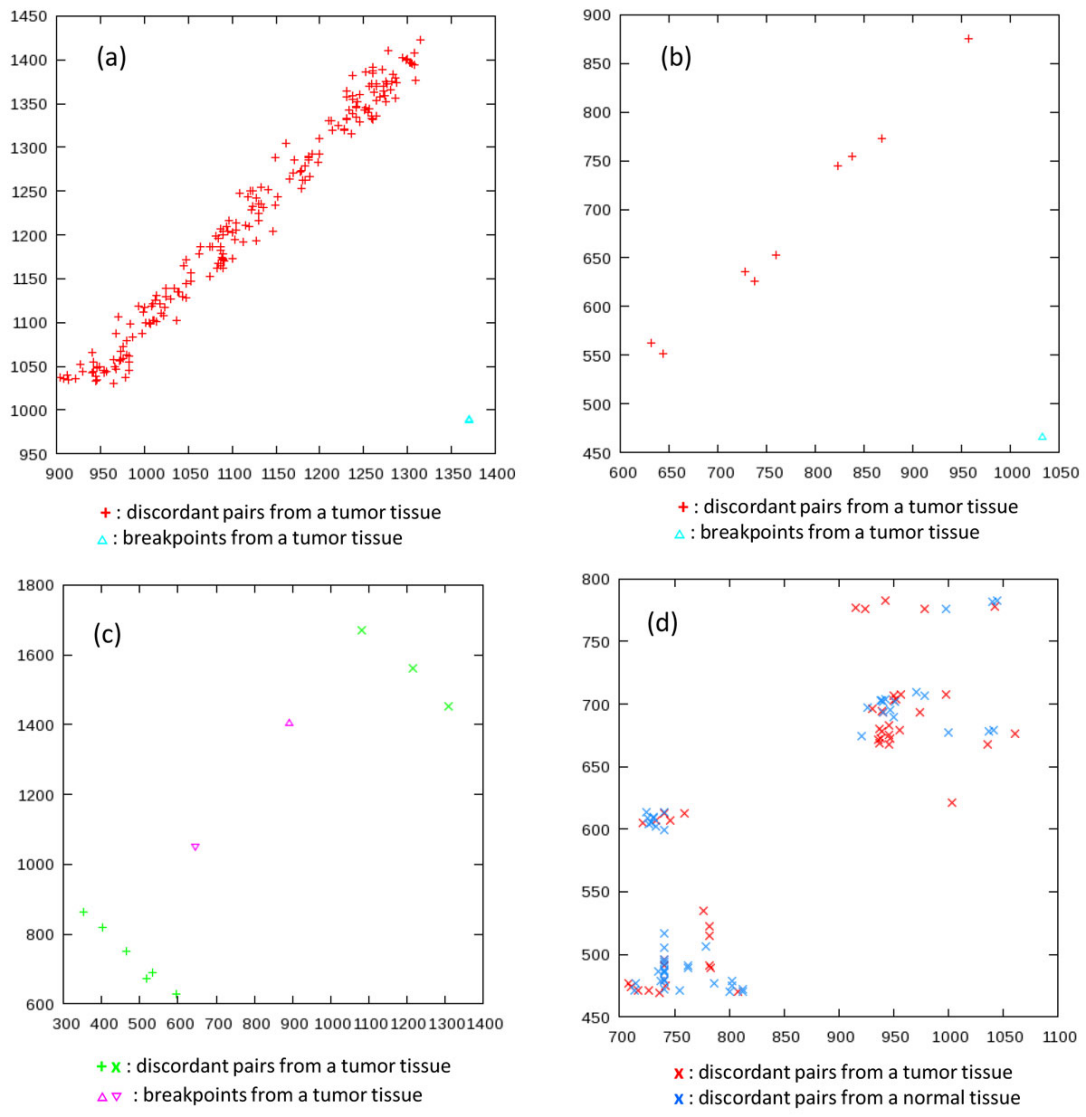
**Examples of two-dimensional clusters of discordant pairs**. (a) A dense cluster associated with a breakpoint; (b) a sparse cluster associated with a breakpoint; (c) a composite cluster associated with two breakpoints; and (d) an indefinitely-shaped cluster not associated with breakpoints.

Figure 5 shows examples of two-dimensional clusters of discordant pairs (represented by + and *×*) along with the associated breakpoints (represented by Δ and ∇).

Clusters (a) to (c) consist of discordant pairs only from the tumor tissue, namely, not from the normal tissue, and they are therefore somatic. Clusters (a) and (b) are associated with a single breakpoint; the discordant pairs are distributed in a belt as described in the previous section. Cluster (a) is dense, seemingly resulting from copy-number amplification, and cluster (b) is sparse, indicating low concentration in the heterogeneous tumor sample. Cluster (c) is composite, associated with two nearby breakpoints; the discordant pairs are distributed along two belts corresponding to the breakpoints. Cluster (d) is not associated with any breakpoints. Discordant pairs from the tumor and normal tissues are mixed; and they are distributed not in belts in the diagonal or anti-diagonal direction but in indefinite shapes. It seems to stem from two reasons: (i) variations and resulting accidental hits to similar sequences far apart, and (ii) fluctuations of the sonicated positions and the insert lengths.

#### Multivalued edit-distance function around a breakpoint

**Figure 6:**
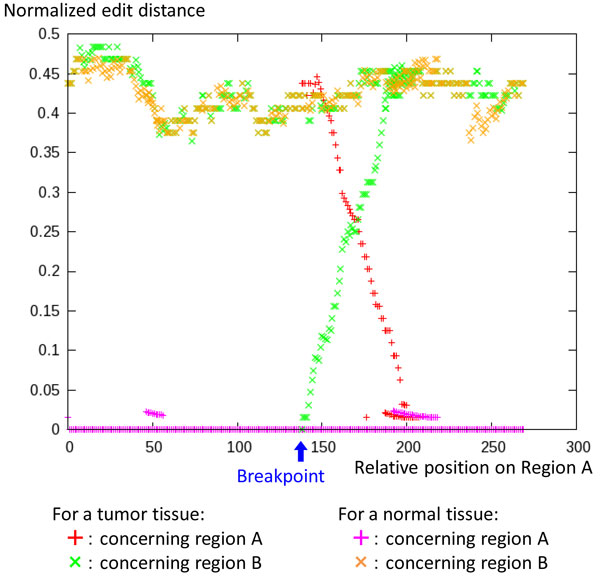
**Example of multivalued edit-distance functions**. Existence of a somatic breakpoint as well as its precise position are indicated by the multivalued edit-distance functions.

Figure 6 shows an example of the multivalued edit-distance function around a breakpoint in the case of real biological data. The edit distance is normalized by the length of extension. The edit distance for the tumor sample shows a characteristic change as described in the previous section. It indicates the existence of a breakpoint as well as its precise position. However, the edit distance for the normal sample does not show any such characteristic change. Therefore, the breakpoint is surely somatic.

### Performance of breakpoint detection

To examine the performance of breakpoint detection, simulation data with known artificial rearrangements were employed. They were generated as follows.

An artificial normal genome sequence was obtained by introducing random SNPs at 0.1% rate into the human reference genome, hg19. An artificial cancer genome sequence was obtained from the artificial normal genome sequence by randomly introducing 40 rearrangements of each of six types: insertion, deletion, inversion, tandem duplication, and intra-chromosomal and inter-chromosomal translocations. In total, 480 breakpoints were introduced by these rearrangements. For each type of rearrangement, the lengths of affected subsequences were chosen uniformly ranging from 300 bp to 30 Kbp. Artificial paired-end short-read WGS data sets were randomly generated from the artificial normal and cancer genome sequences approximately at 40-times (or 20-times) coverage; each data set amounted to about 115 Gbp (or 58 Gbp). The read lengths were set to 90 bp, and the insert lengths were chosen so that they were distributed according to the Gaussian distribution with mean 760 bp and standard deviation 45 bp. These parameters for generating WGS data sets were chosen according to real biological data [23]. The WGS data sets of artificial heterogeneous cancer samples at different cancer purities and of the same size were generated by mixing the artificial WGS data sets from the artificial normal and cancer genome sequences.

Table 1 shows the sensitivity of breakpoint detection for different cancer purities at 40-times coverage. Sensitivity begins to decrease significantly when the tumor purity drops below around 20%. The numbers of false detections in these data sets are 0, 2, 1, 1, and 2 (not shown in the table); thus, the false detection rates are very low (less than 1%).

**Table 1:** Sensitivity of breakpoint detection at 40-times coverage.

Data set	Cancer purity	Number of breakpoints		Sensitivity
		**Predicted**	**Detected**	

sim10	100%	457	455	95%

sim05	50%	455	452	94%

sim03	30%	456	446	93%

sim02	20%	454	433	90%
sim01	10%	445	356	74%

Simulation data with known artificial rearrangements are employed for evaluation of the proposed method. The third and fourth columns indicate the number of predicted breakpoint regions and the number of detected breakpoints, respectively. The last column is the ratio of the fourth column to all (480) breakpoints.

The sensitivity of breakpoint detection is somewhat different for different types of rearrangement (Table 2). The sensitivity is lowest (90%) in the case of tandem duplications. The same copies of a sequence generated by a tandem duplication tend to mislead the mapping tools, resulting in loss of discordant pairs.

**Table 2:** Performance of breakpoint detection for different types of rearrangement in sim10 at 40-times coverage.

Type	Number of breakpoints		Sensitivity
	**Total**	**Detected**	

Insertions	80	76	95%

Deletions	40	38	95%

Inversions	80	78	98%

Intra-chrom. trans.	120	114	95%
Inter-chrom. trans.	120	113	94%

Tandem dup.	40	36	90%

The second column is the number of breakpoints associated with each type of rearrangement. The third column is the number of detected breakpoints corresponding to each type. The last column is the ratio of the third column to the second column. Intra-chrom. trans.: intra-chromosomal translocations, Inter-chrom. trans.: inter-chromosomal translocations, Tandem dup.: tandem duplications.

Table 3 compares the performance of breakpoint detection on simulated WGS data with that of other tools. The proposed method is comparable in terms of sensitivity and has much smaller false-detection rate.

**Table 3:** Comparisons of performance of proposed method and other tools.

Method	Target	Depth	Purity	Sensitivity	FDR
This study	Misc	×40	20%	90%	<1%

		×20	20%	74%	<1%

PRISM[15]	D	×20	100%	80%	5%

LUMPY[17]	D	×40	20%	96%	4%

		×20	20%	77%	4%

CREST[16]	D,T	×40	50%	73-78%	3%

Targeted types of rearrangement and features of simulated WGS data (including the sequencing depth) differ from tool to tool. D: deletions, T: tandem duplications, Misc: insertions, deletions, inversions, tandem duplications, and intra-chromosomal and inter-chromosomal translocations, FDR: false-detection rate.

### Application to real biological data

The proposed method was applied to real biological data, and the results were compared with published known results [23]. The real biological data were taken from publicly available WGS data of patients with HBV (Hepatitis B virus)-associated HCC (hepatocellular carcinoma) in a study ERP001196 in the NCBI SRA. The matched tumor and normal samples were taken from excised tumor liver tissues and surrounding normal liver tissues. These normal tissues did not seem to be truly normal, unlike blood samples, because they were likely to be exposed to HBV infection for a long time with potential damage to the genomic DNA. Two kinds of libraries with long and short insert lengths (approximately, 800 and 200 bp) were used, and the read lengths were 90 bp. Total amount of data in each sample ranged approximately from 100 to 120 Gbp.

Tables 4(a) and (b) show the numbers of somatic breakpoints in different patients detected by the proposed method and those reported by Banet *et al*. [23] using CREST [16]. The intra-chromosomal and inter-chromosomal somatic breakpoints are separately counted in Tables (a) and (b), respectively. Detailed lists of detected somatic breakpoints are given in Additional file 1.

**Table 4:** Comparison of detected somatic breakpoints with those reported by Banet *et al.*

(a) Number of intra-chromosomal somatic breakpoints					
**patient ID**	**predicted**	**detected**	**known**	**agree**	**rate**

22	51	17	13	6	46%

23	67	49	43	27	63%

39	113	92	91	72	79%

43	94	89	77	58	75%

45	48	42	30	21	70%

64	123	113	91	70	77%

71	11	10	9	2	22%

81	8	7	12	6	50%

90	44	39	29	19	66%

117	242	219	158	146	92%

126	55	17	11	7	64%

145	83	72	55	50	91%

172	179	139	135	91	67%

198	88	53	65	37	57%

207	72	65	58	49	84%

268	75	49	41	31	76%

Total	1353	1072	918	692	75%

(b) Number of inter-chromosomal somatic breakpoints					

**patient ID**	**predicted**	**detected**	**known**	**agree**	**rate**

22	10	8	1	0	0%

23	14	12	5	2	40%

39	34	30	14	8	57%

43	19	18	9	5	56%

45	15	12	12	9	75%

64	14	11	9	3	33%

71	12	8	7	0	0%

81	5	4	4	0	0%

90	7	6	6	1	17%

117	17	15	6	3	50%

126	4	4	3	0	0%

145	41	37	20	14	70%

172	33	25	8	3	38%

198	8	7	1	0	0%

207	6	6	7	1	14%

208	9	9	7	3	43%

Total	248	212	119	52	44%

[23].

The second and third columns are the number of predicted somatic breakpoint regions and the number of detected somatic breakpoints, respectively, for each patient. The fourth column is the number somatic breakpoints reported by [23]. The fifth column is the number of breakpoints that are counted in the third column and in the forth column (i.e., the intersection of both columns). The last column is the ratio of the fifth column to the fourth column. The intra-chromosomal and inter-chromosomal somatic breakpoints are separately counted in Tables (a) and (b), respectively. Detailed lists of detected somatic breakpoints are given in Additional file 1. As for patient ID 71, evidential materials for detected somatic intra-chromosomal breakpoints are given in Additional file 3.

As for the intra-chromosomal events, the agreement rate between the breakpoints detected by the proposed method and those reported by Banet *et al*. is generally much smaller than the sensitivity of the proposed method evaluated above. Accordingly, the case (patient ID 71) with the lowest agreement rate was further investigated.

Among nine somatic breakpoints reported by Banet *et al*., only two were detected by the proposed method; five were actually found in both the tumor and normal samples, and they were therefore not tumor-specific; one was a rearrangement in a small distance (90 bp) and was actually missed by the proposed method; and one remained unclear, and no clues were found. The evidential materials are given in Additional file 2. Likewise, in other cases, many of the somatic breakpoints reported by Banet *et al*. but not detected by the proposed method were in fact not tumor-specific.

On the other hand, somatic breakpoints not reported by Banet *et al*. were detected by the proposed method. They are roughly 0.5 times more abundant than ones in agreement. If the low false-detection rate evaluated above is applicable in these cases, the extra breakpoints are new findings not reported previously. As for the same patient ID 71 as above, evidential materials for extra somatic breakpoints are given in Additional file 3.

The top four patients with the largest numbers of detected intra-chromosomal somatic breakpoints, namely, those with ID 117, 172, 64, and 39, are reported to have chromothripsis [23], which is known to induce hundreds of very complicated intra-chromosomal genomic rearrangements [11].

As for the inter-chromosomal events, the agreement rate between the breakpoints detected by the proposed method and those reported by Banet *et al*. is even lower. The difference in the agreement rate between the intra-chromosomal and inter-chromosomal events are far larger than the difference between the evaluated sensitivity of the intra-chromosomal and interchromosomal translocations (Table 2). The somatic breakpoints detected by the proposed method but not reported by Banet *et al*. are roughly three times as many as the ones in agreement. They are likely to be new findings not reported previously, if the evaluated low false-detection rate of the proposed method is also applicable in these cases.

Although the inter-chromosomal events were generally much fewer than the intra-chromosomal events, tens of inter-chromosomal events were detected in some patients. These events correspond to a feature of chromoplexy that are known to induce tens of very complicated inter-chromosomal rearrangements [12,13].

### Computational time and memory usage

A dual-CPU PC server (Intel Xeon, E5-2680, 256 GB memory) was used in the above computational experiments, prior to which the following data were prepared: Burrows-Wheeler transforms of WGS data [7] and discordant pairs extracted from BAM files (mapping results onto the human reference genome) obtained by using BWA [4] and SAMtools [19].

The method for predicting breakpoint regions and that for detecting breakpoints therein were implemented in Perl and C++, respectively. Their computational times were roughly proportional to the number of discordant pairs and the number of predicted regions, respectively. The maximum memory usage was dominated by the size of the Burrows-Wheeler transform data and was roughly proportional to the number of base pairs in the WGS data.

As for the case of patient ID 64, with about 13 million (the largest number among the above-mentioned patients) discordant pairs, the time for predicting breakpoint regions was 448 seconds, and the time for detecting breakpoints was 375 seconds (including 201 seconds for loading the Burrows-Wheeler transform data into memory); the maximum memory usage for the WGS data of 234 Gbp (i.e., total size for matched tumor and normal samples) was 73 GB.

## Conclusions

The BWT of short-read data, serving as a lossless dictionary of reads, enables sensitive analysis of genomic rearrangements in heterogeneous cancer-genome samples when used in conjunction with breakpoint-region predictions. Breakpoint regions are predicted by means of a conjugate transformation of an empirical distribution of discordant pairs. The breakpoint regions are efficiently examined by using the BWT of reads and a fast dynamic programming method, and the break-points are detected by using the multivalued edit-distance functions, and their precise positions are determined. The proposed method was demonstrated to actually work on real biological data by using publicly available WGS data of cancer patients. It achieved comparable sensitivity to existing tools and much lower false-detection rate when applied to simulation data with known artificial rearrangements. Moreover when applied to publicly available WGS data of cancer patients, it detected many somatic breakpoints that were not reported previously in the literature.

## Competing interests

The authors declare that they have no competing interests.

## Authors' contributions

All authors designed the study and examined the results. KK carried out the computational experiments and drafted the manuscript. AK supervised the study and helped draft the manuscript. All authors read and approved the final manuscript.

## Supplementary Material

Additional file 1 **Detailed list of breakpoint detection for real biological WGS data**. The data are publicly available WGS data of patients with HBV (hepatitis B virus)-associated HCC (hepatocellular carcinoma) in a study ERP001196 in the NCBI SRA [23]. Click here for file

Additional file 2 Evidential materials (1) in the analysis of Patient ID 71. The disagreement between the results by the proposed method and those reported by Banet *et al*. [23] is the greatest in case of patient ID 71. Evidential material is given for each event reported by Banet *et al*. Click here for file

Additional file 3 **Evidential materials (2) in the analysis of Patient ID 71**. For the same patient, evidential materials for detected somatic intra-chromosomal breakpoints are given. Click here for file

## Declarations

Publication charges for this article have been funded by Hitachi, Ltd.

This article has been published as part of *BMC Bioinformatics *Volume 16 Supplement 18, 2015: Joint 26th Genome Informatics Workshop and 14th International Conference on Bioinformatics: Bioinformatics. The full contents of the supplement are available online at http://www.biomedcentral.com/bmcbioinformatics/supplements/16/S18.
