# Supplementary material for: Analysis of genomic rearrangements by using the Burrows-Wheeler transform of short-read data
Source: BMC Bioinformatics. 2015 Dec 9;16(Suppl 18):S5. doi: 10.1186/1471-2105-16-S18-S5 (PMC4708002; doi:10.1186/1471-2105-16-S18-S5)
Supplement: Additional file 2 — Evidential materials (1) in the analysis of Patient ID 71. The disagreement between the results by the proposed method and those reported by Banet et al. [23] is the greatest in case of patient ID 71. Evidential material is given for each event reported by Banet et al. [file 1471-2105-16-S18-S5-S2.pdf]

# Additional File 2

- Discordant pairs associated with a breakpoint are distributed along two belts corresponding  to long and short (approximately, 800 and 200 bp) insert lengths.
- +, ×, +, × : discordant pair from a tumor sample.
- +, ×, +, × : discordant pair from a normal sample.
- ○ :  breakpoint reported by Banet et al.
- EVENTs are reported by Banet et al.
- EVENT4054, 4055, 4056, 4059, 4060 are not somatic but germline.
- EVENT4058, 4062 are also detected by proposed methods.
- EVENT4061 are not detected by the proposed methods.
- EVENT4057 remains unclear.
- See Additional File 3 for results by the proposed method.

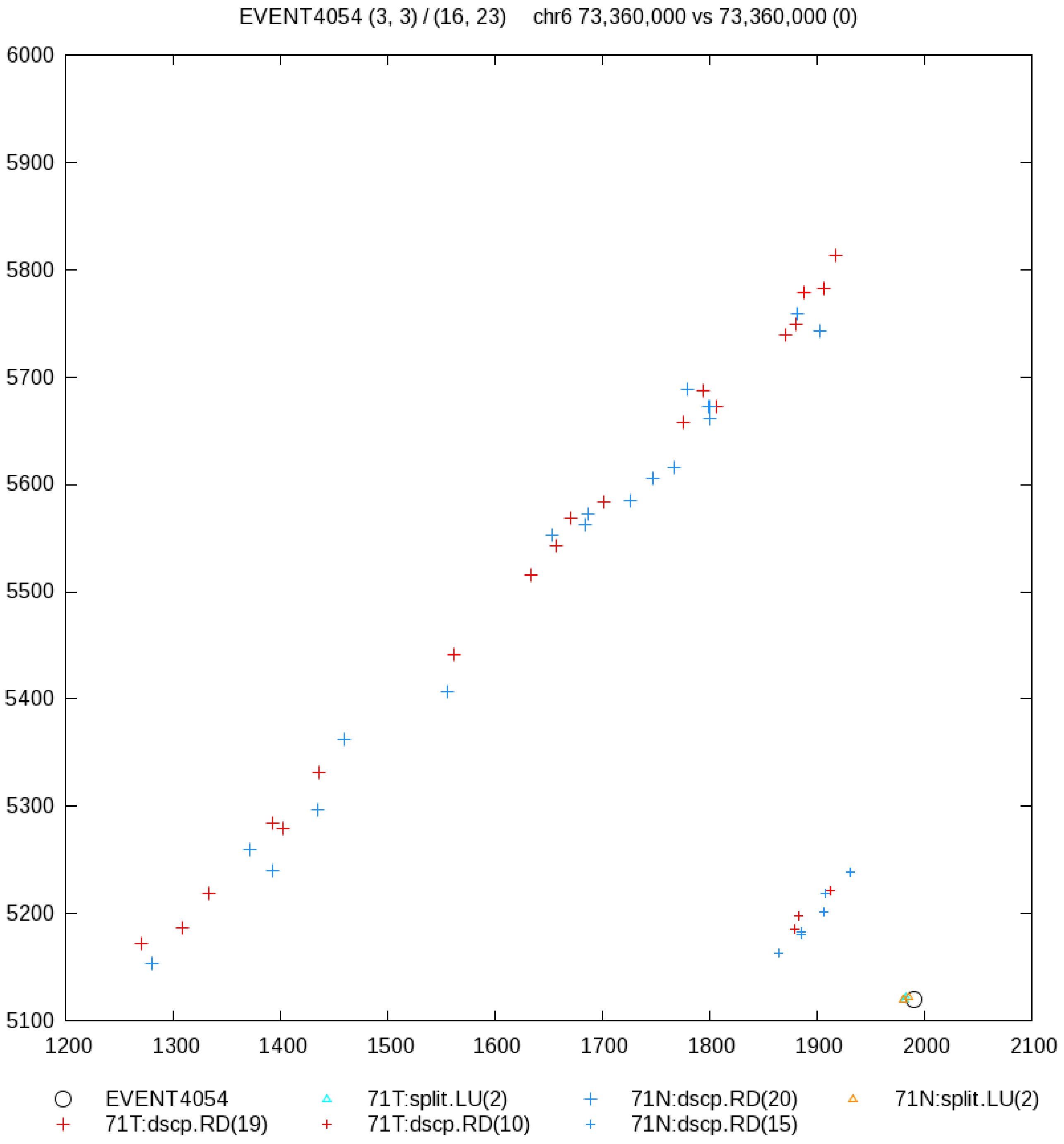
EVENT4054 (3, 3) / (16, 23) chr6 73,360,000 vs 73,360,000 (0)
6000
5900
5800
5700
5600
5500
5400
5300
5200
5100
1200
1300
1400
1500
1600
1700
1800
1900
2000
2100
EVENT4054
71T:split.LU(2)
71N:dscp.RD(20)
71N:split.LU(2)
71T:dscp.RD(19)
71T:dscp.RD(10)
71N:dscp.RD(15)

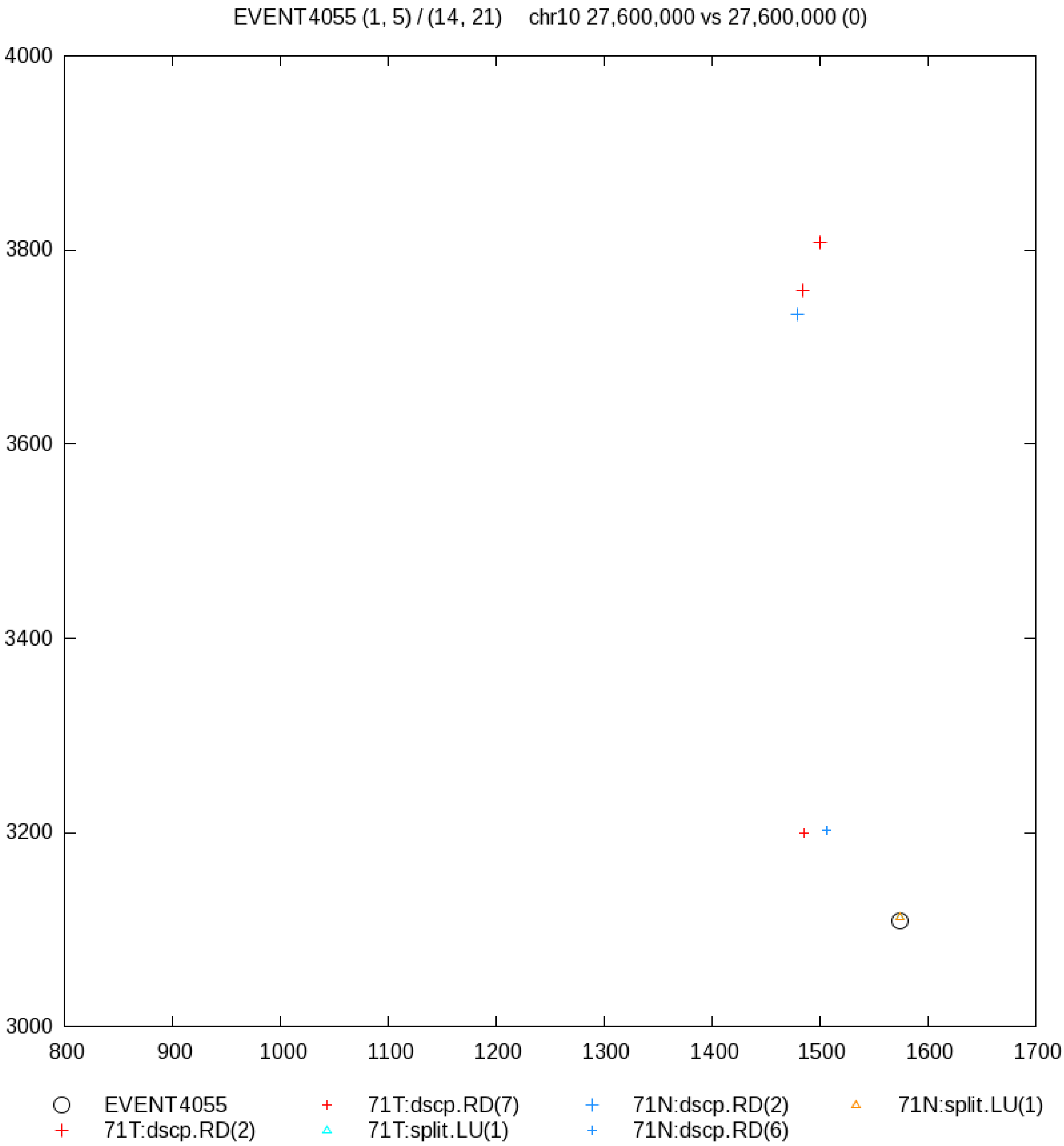

EVENT4055 (1, 5) / (14, 21) chr10 27,600,000 vs 27,600,000 (0)
4000
3800
3600
3400
3200
3000
800
900
1000
1100
1200
1300
1400
1500
1600
1700
EVENT4055
71T:dscp.RD(7)
71N:dscp.RD(2)
71N:split.LU(1)
71T:dscp.RD(2)
71T:split.LU(1)
71N:dscp.RD(6)

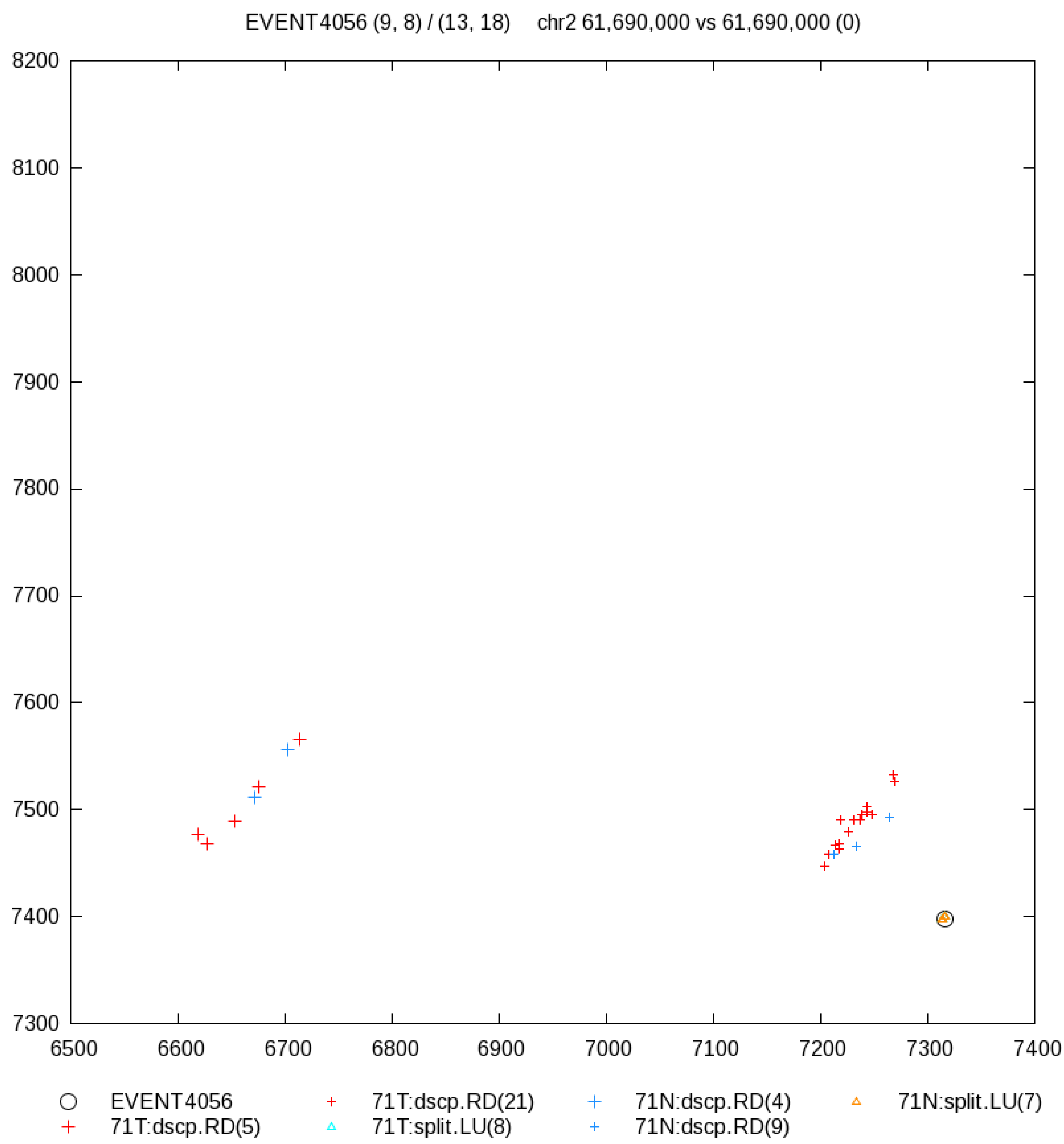

EVENT4056 (9, 8) / (13, 18)     chr2 61,690,000 vs 61,690,000 (0)
8200
8100
8000
7900
7800
7700
7600
7500
7400
7300
6500
6600
6700
6800
6900
7000
7100
7200
7300
7400
EVENT4056
71T:dscp.RD(21)
71N:dscp.RD(4)
71N:split.LU(7)
71T:dscp.RD(5)
71T:split.LU(8)
71N:dscp.RD(9)

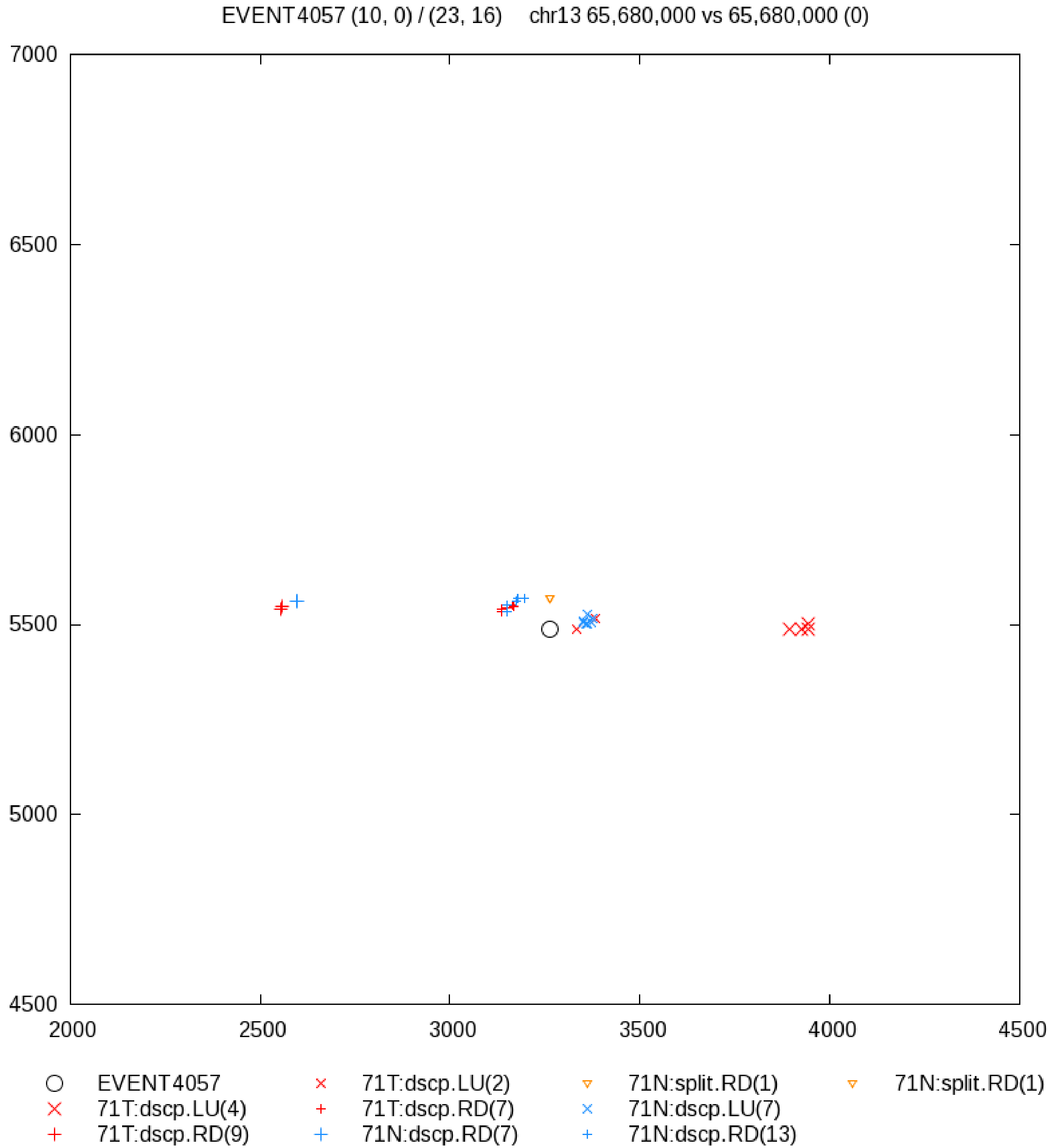

EVENT4057 (10, 0) / (23, 16) chr13 65,680,000 vs 65,680,000 (0)
7000
6500
6000
5500
5000
4500
2000
2500
3000
3500
4000
4500
EVENT4057
71T:dscp.LU(2)
71N:split.RD(1)
71N:split.RD(1)
71T:dscp.LU(4)
71T:dscp.RD(7)
71N:dscp.LU(7)
71T:dscp.RD(9)
71N:dscp.RD(7)
71N:dscp.RD(13)

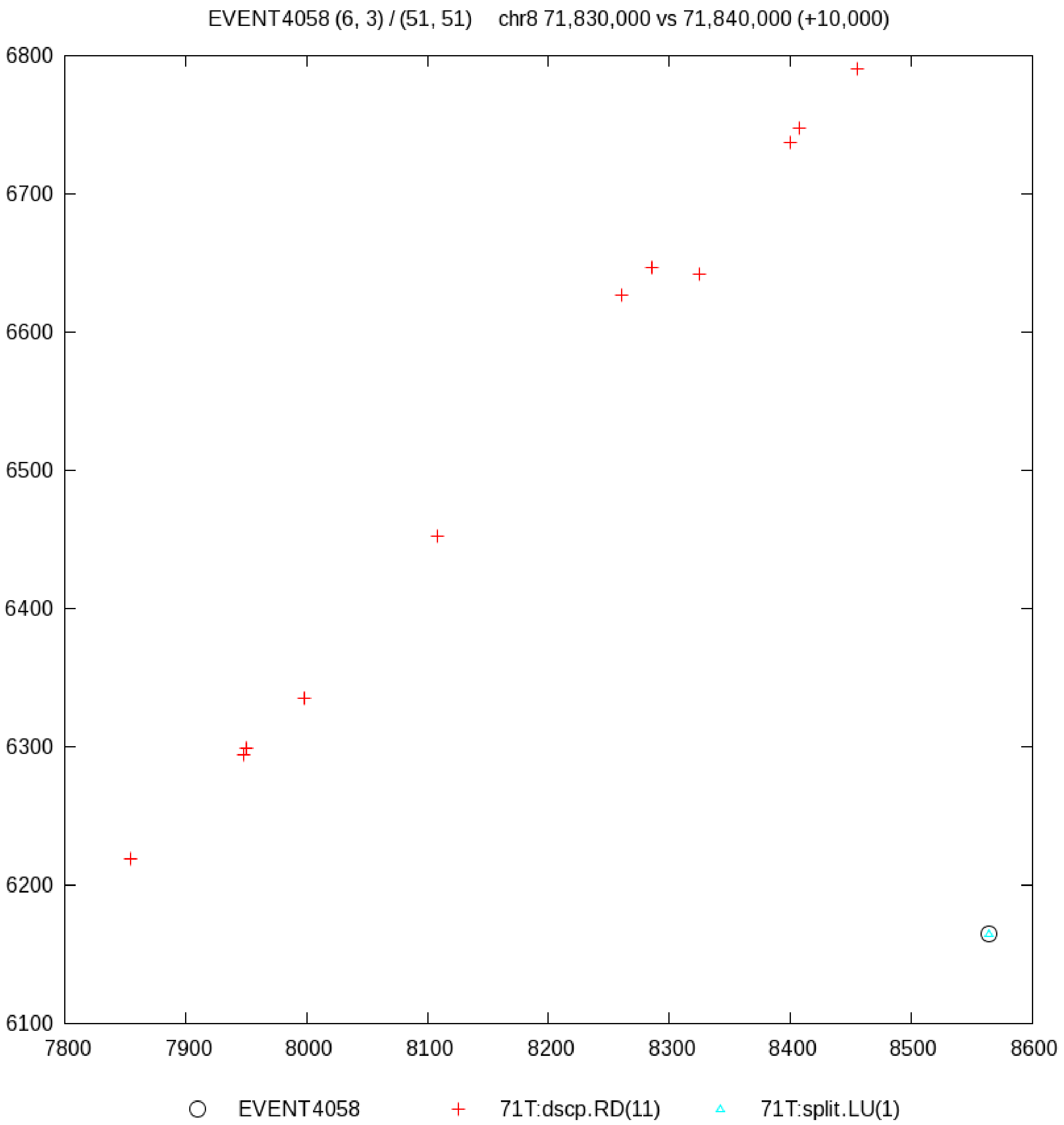
EVENT4058 (6, 3) / (51, 51)     chr8 71,830,000 vs 71,840,000 (+10,000)
6800
6700
6600
6500
6400
6300
6200
6100
7800
7900
8000
8100
8200
8300
8400
8500
8600
EVENT4058
71T:dscp.RD(11)
71T:split.LU(1)

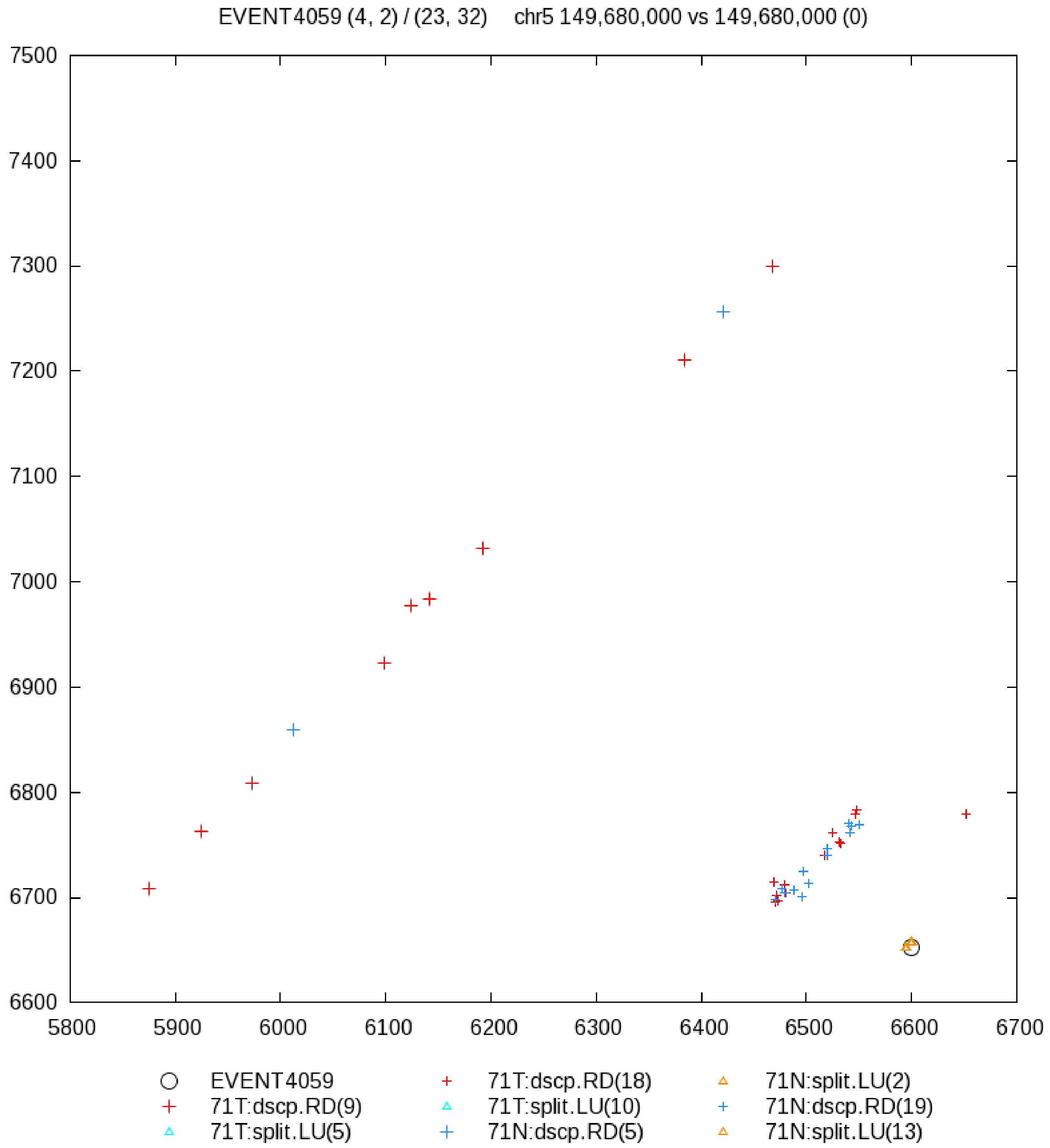
EVENT4059 (4, 2) / (23, 32) chr5 149,680,000 vs 149,680,000 (0)
7500
7400
7300
7200
7100
7000
6900
6800
6700
6600
5800
5900
6000
6100
6200
6300
6400
6500
6600
6700
EVENT4059
71T:dscp.RD(9)
71T:split.LU(5)
71T:dscp.RD(18)
71T:split.LU(10)
71N:dscp.RD(5)
71N:split.LU(2)
71N:dscp.RD(19)
71N:split.LU(13)

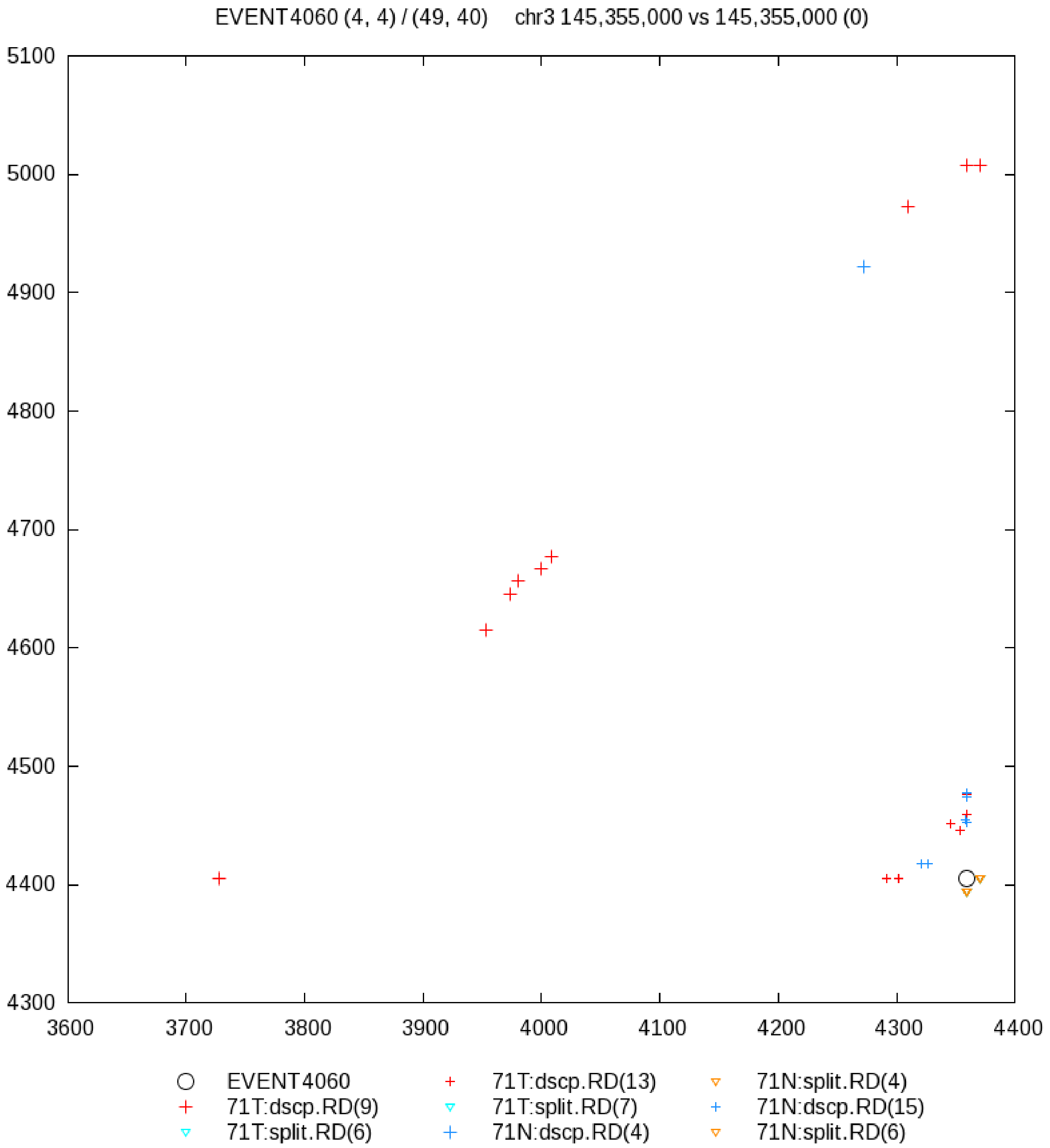
EVENT4060 (4, 4) / (49, 40) chr3 145,355,000 vs 145,355,000 (0)
5100
5000
4900
4800
4700
4600
4500
4400
4300
3600
3700
3800
3900
4000
4100
4200
4300
4400
EVENT4060
71T:dscp.RD(9)
71T:split.RD(6)
71T:dscp.RD(13)
71T:split.RD(7)
71N:dscp.RD(4)
71N:split.RD(4)
71N:dscp.RD(15)
71N:split.RD(6)

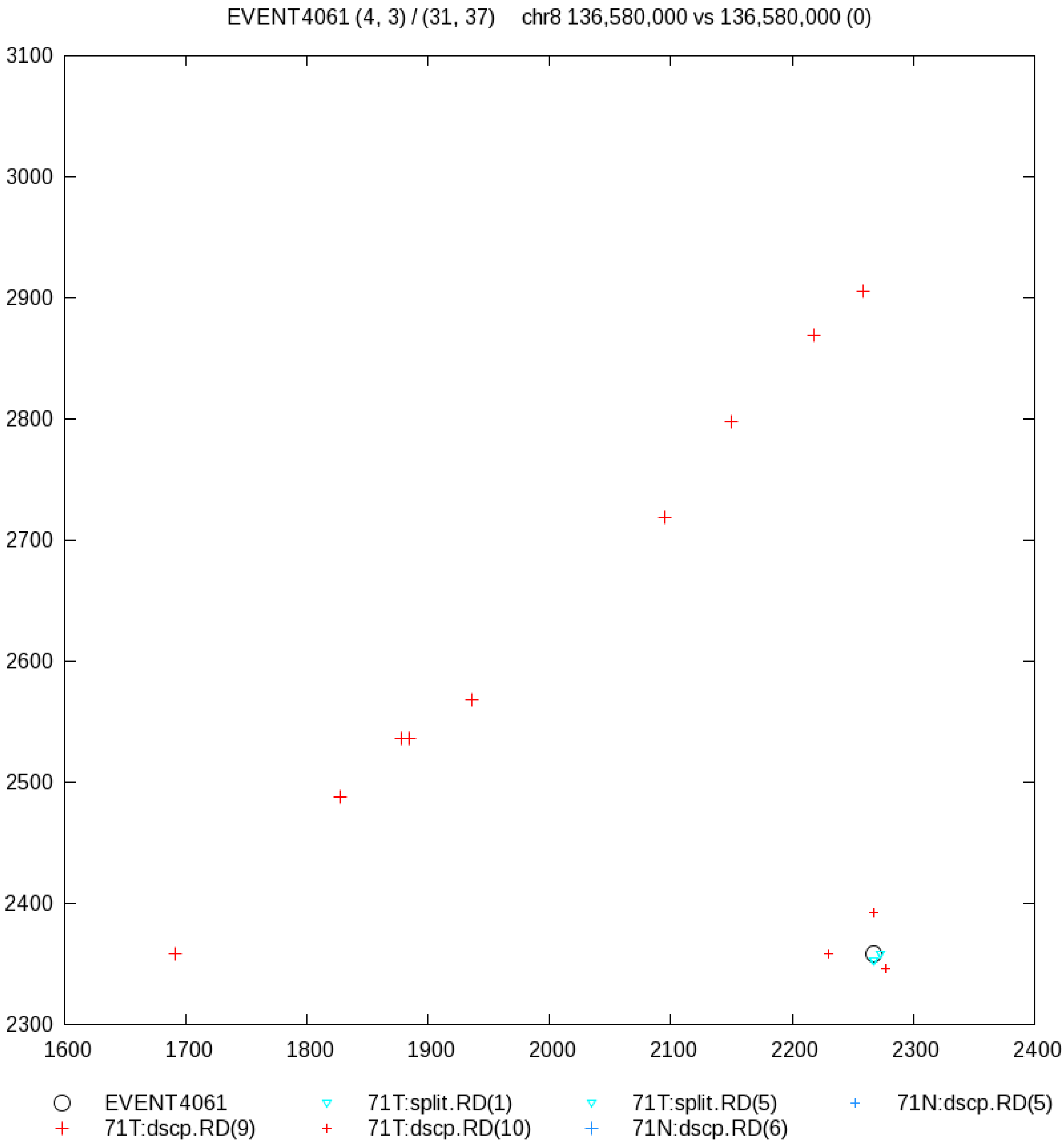
EVENT4061 (4, 3) / (31, 37) chr8 136,580,000 vs 136,580,000 (0)
3100
3000
2900
2800
2700
2600
2500
2400
2300
1600
1700
1800
1900
2000
2100
2200
2300
2400
EVENT4061
71T:split.RD(1)
71T:split.RD(5)
71N:dscp.RD(5)
71T:dscp.RD(9)
71T:dscp.RD(10)
71N:dscp.RD(6)

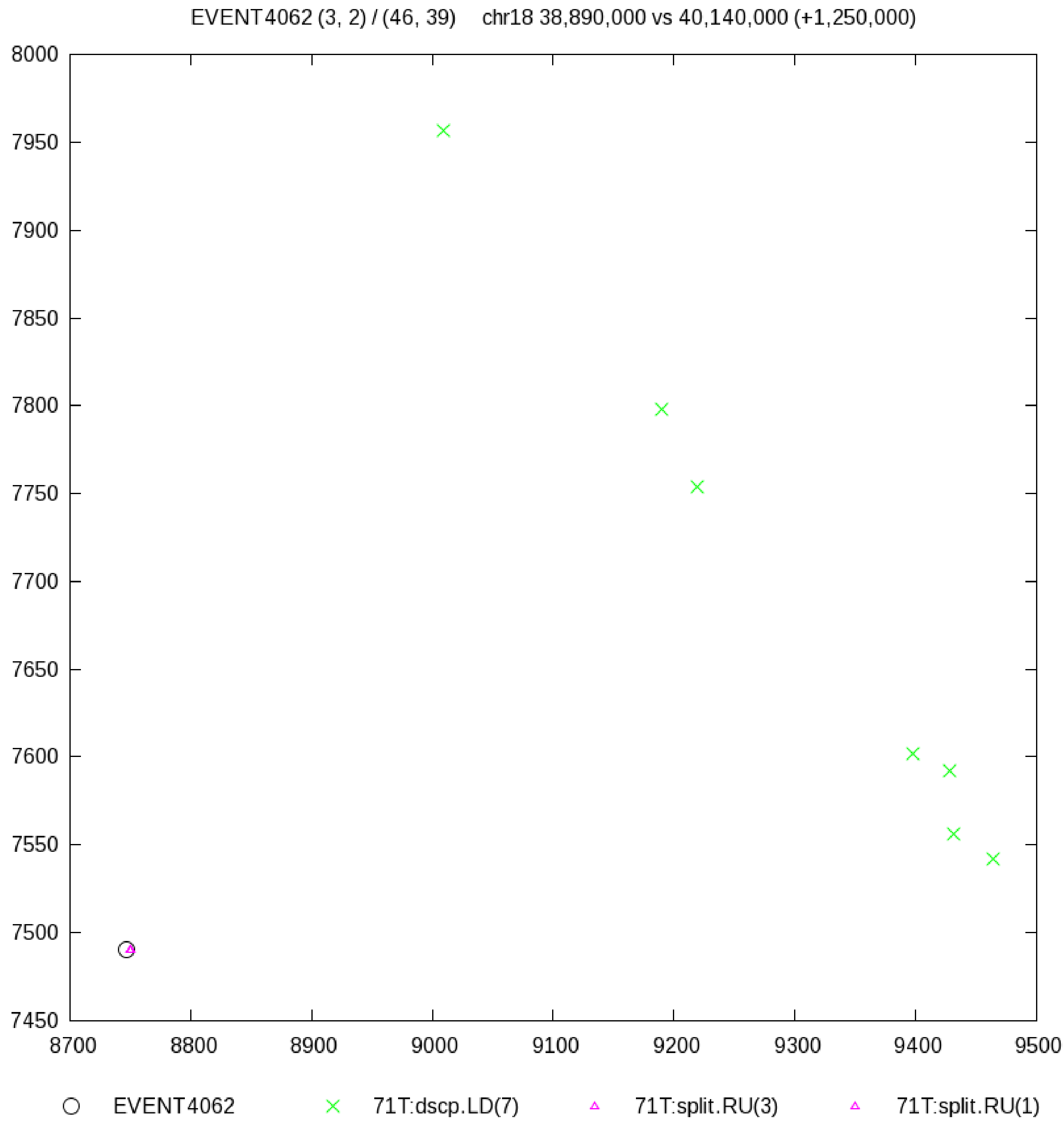

EVENT4062 (3, 2) / (46, 39) chr18 38,890,000 vs 40,140,000 (+1,250,000)
8000
7950
7900
7850
7800
7750
7700
7650
7600
7550
7500
7450
8700
8800
8900
9000
9100
9200
9300
9400
9500
EVENT4062
71T:dscp.LD(7)
71T:split.RU(3)
71T:split.RU(1)
