# Supplementary material for: Analysis of genomic rearrangements by using the Burrows-Wheeler transform of short-read data
Source: BMC Bioinformatics. 2015 Dec 9;16(Suppl 18):S5. doi: 10.1186/1471-2105-16-S18-S5 (PMC4708002; doi:10.1186/1471-2105-16-S18-S5)

Additional File 3

- Discordant pairs associated with a breakpoint are distributed along two belts corresponding to long and short (approximately, 800 and 200 bp) insert lengths.
- +, ×, +, × : discordant pair from a tumor sample.
- +, ×, +, × : discordant pair from a normal sample.
- ○ : breakpoint reported by Banet et al.
- 11 intra-chromosomal somatic clusters (from 0000 to 0010) are found in Patient 71.
- All clusters, except for 0008, has a unique somatic breakpoints.
- Cluster 0008 is not associated with breakpoints.

- Point styles in plotting multi-valued edit-distance functions:

For a tumor tissue:

+ : concerning region A

× : concerning region B

For a normal tissue:

+ : concerning region A

× : concerning region B

Cluster: 0000

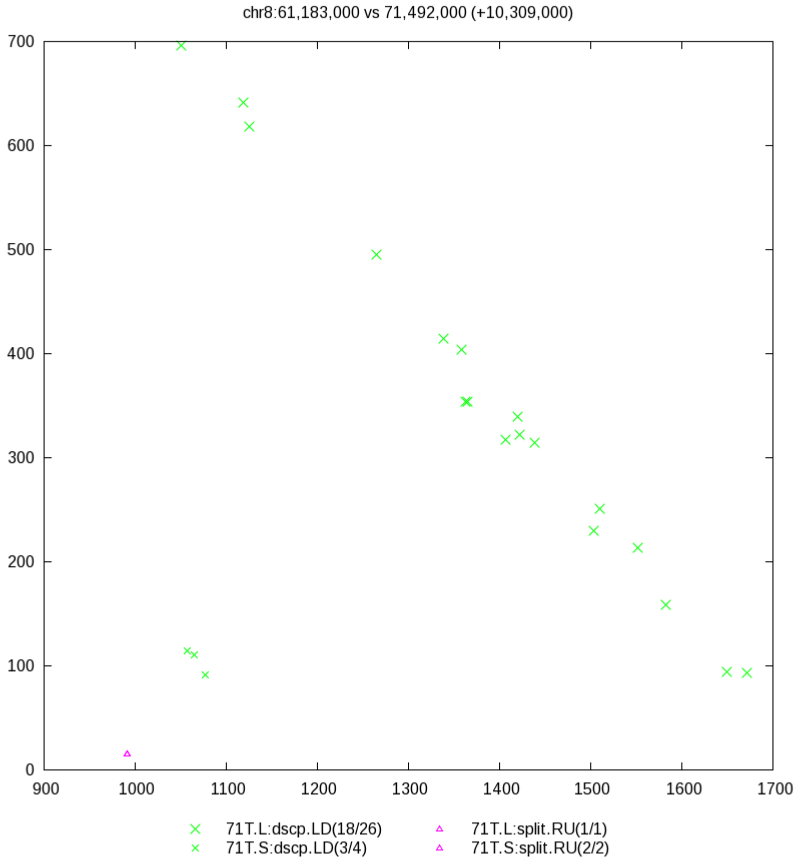

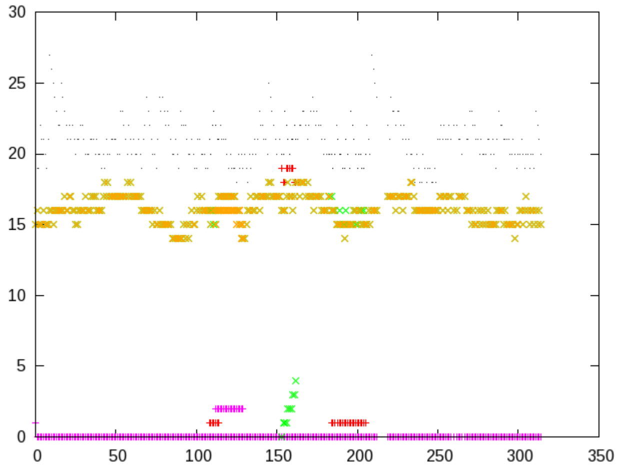

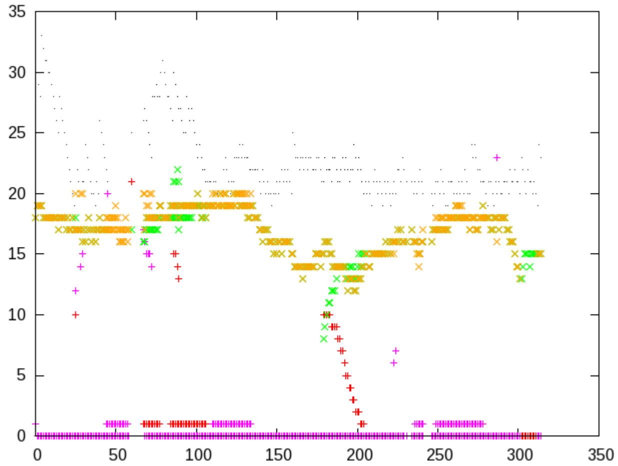

Cluster: 0001

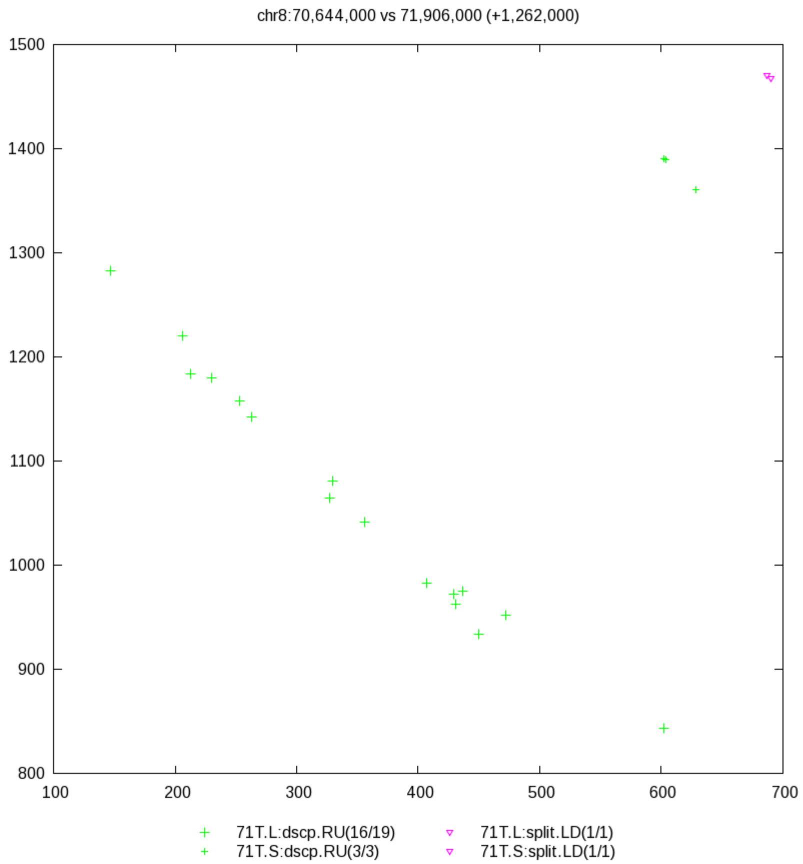

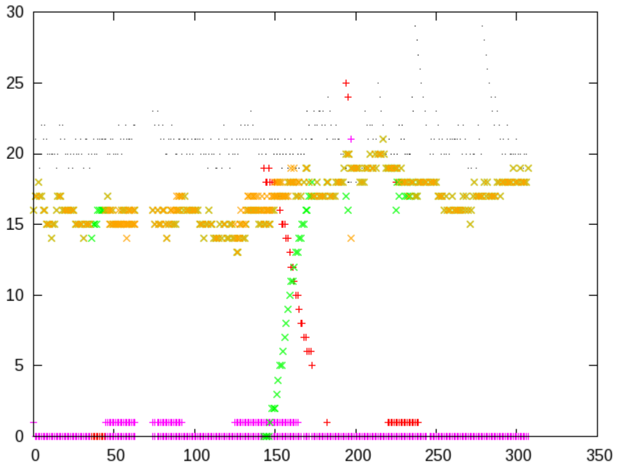

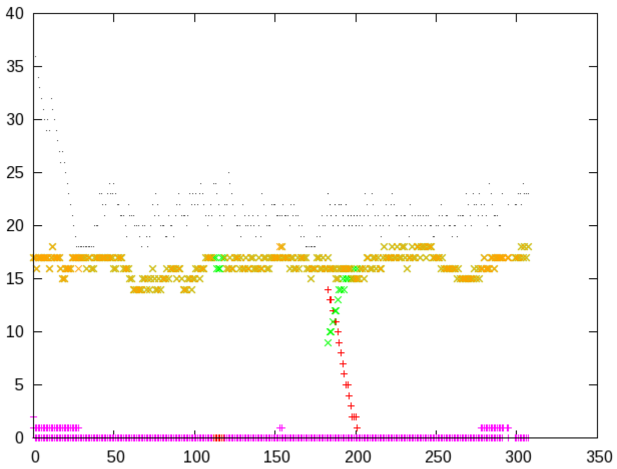

Cluster: 0002

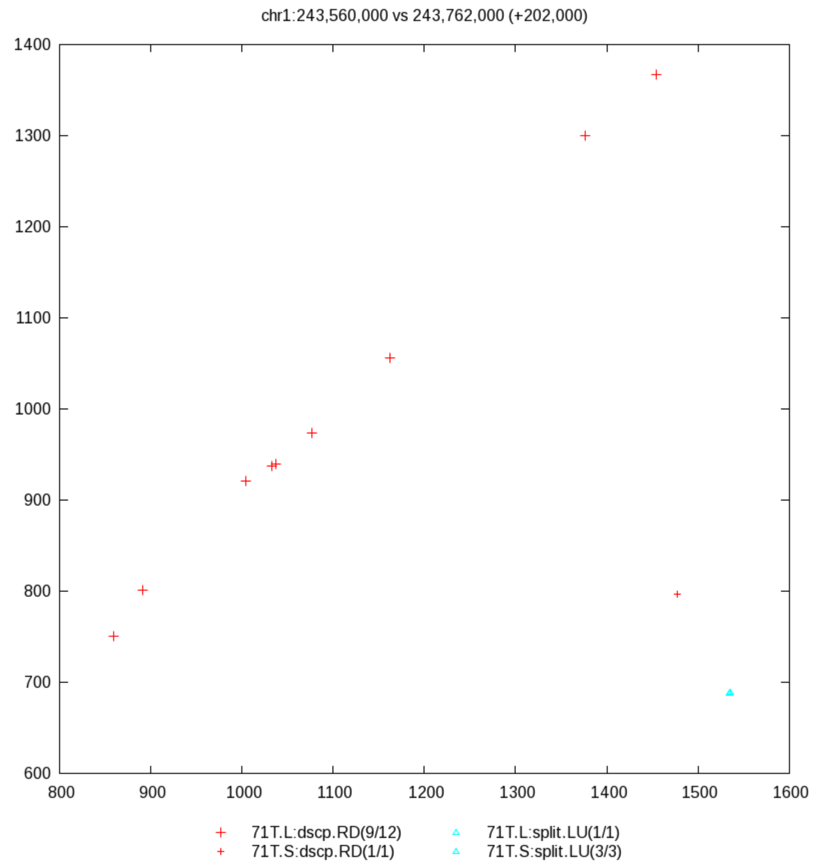

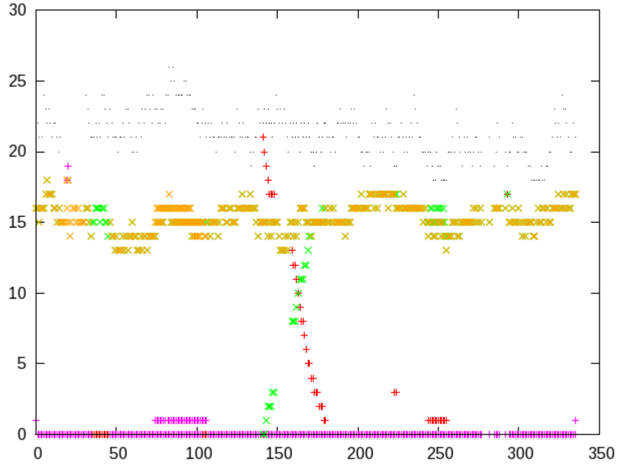

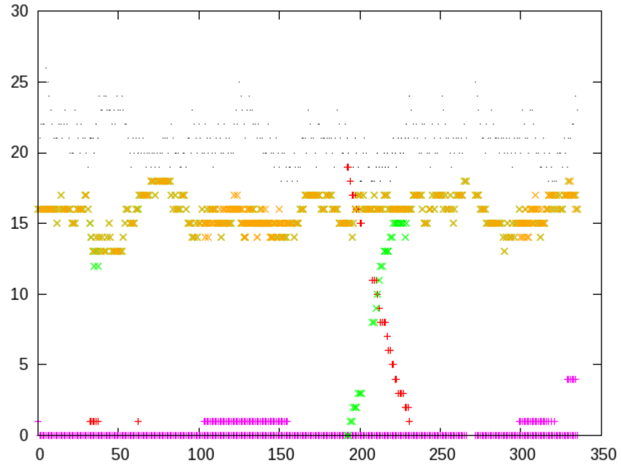

Cluster: 0003

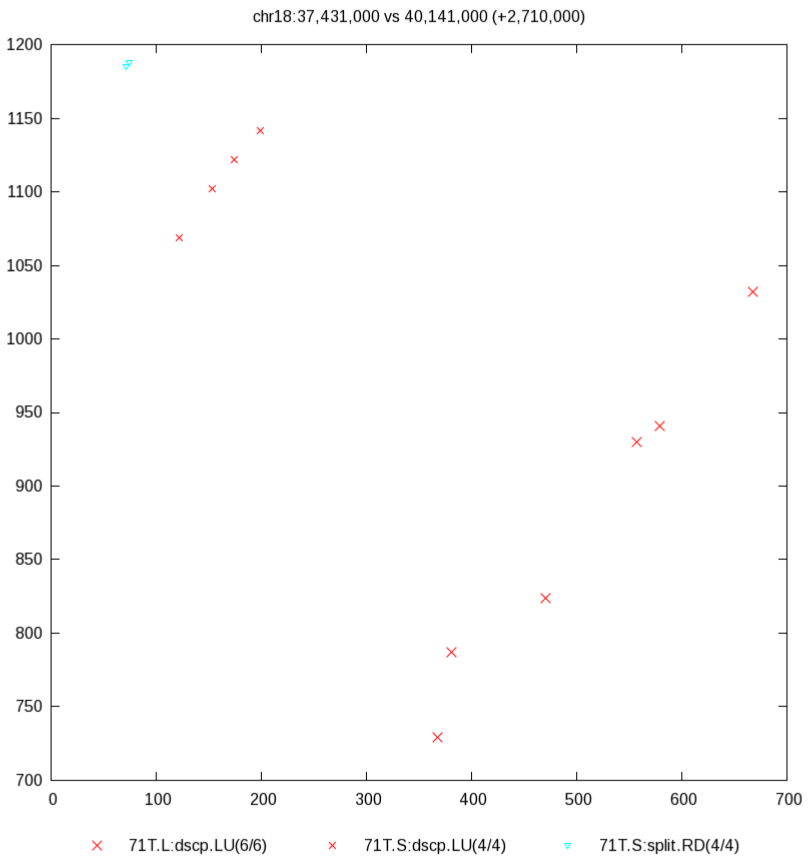

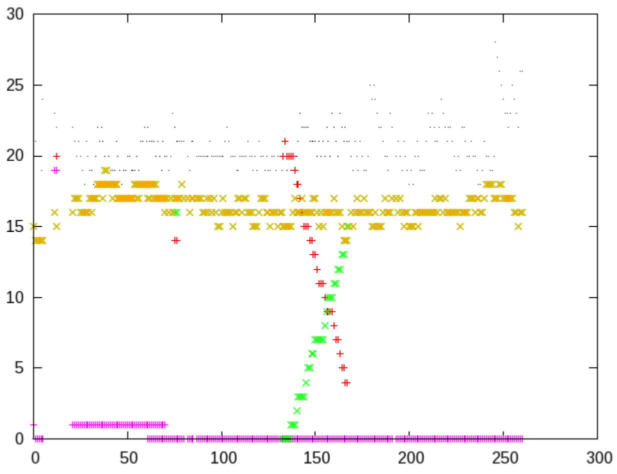

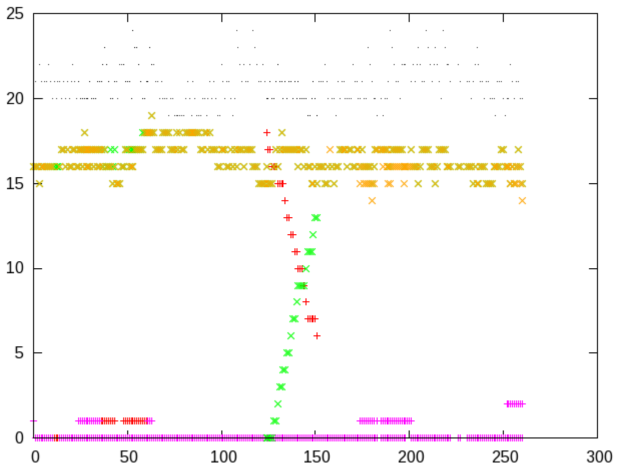

Cluster: 0004

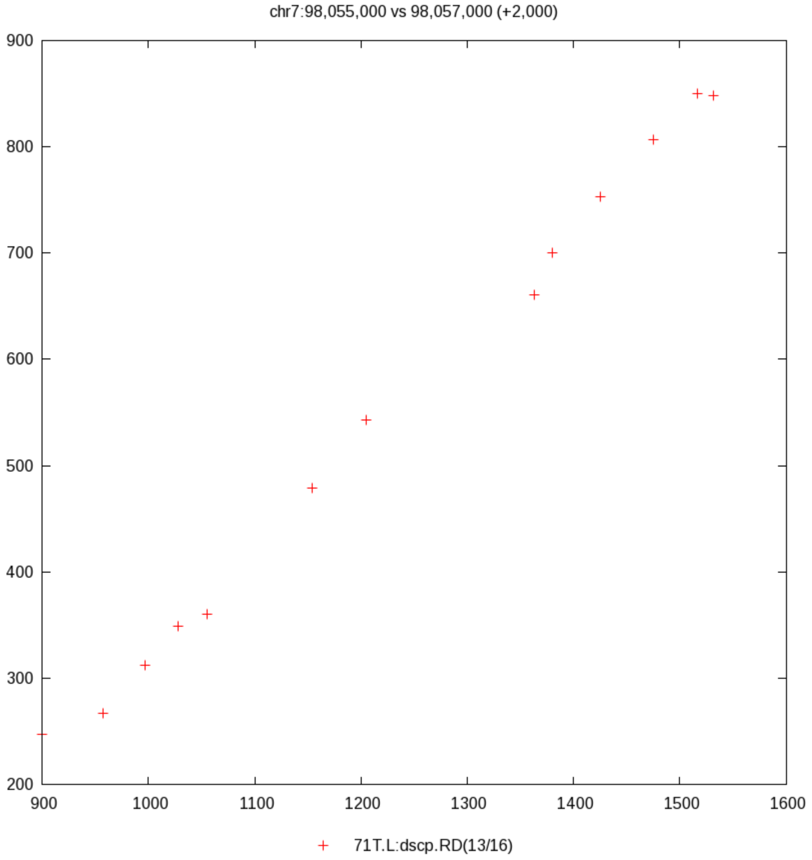

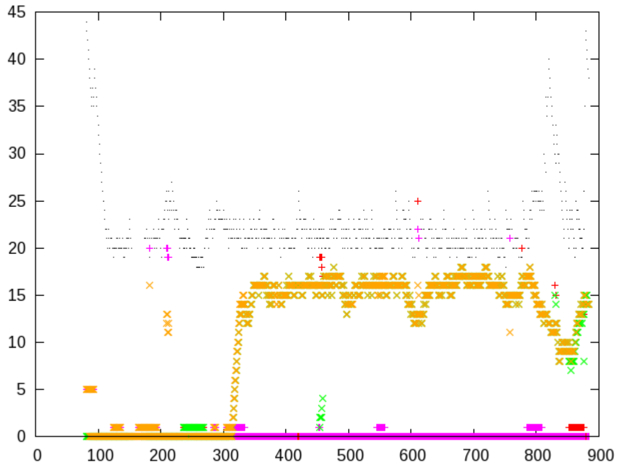

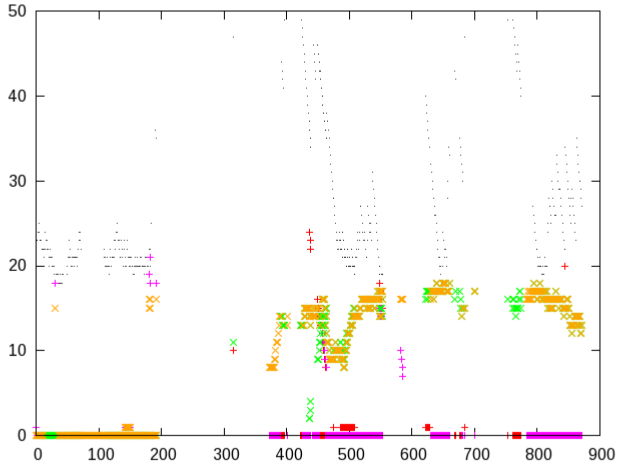

Cluster: 0005

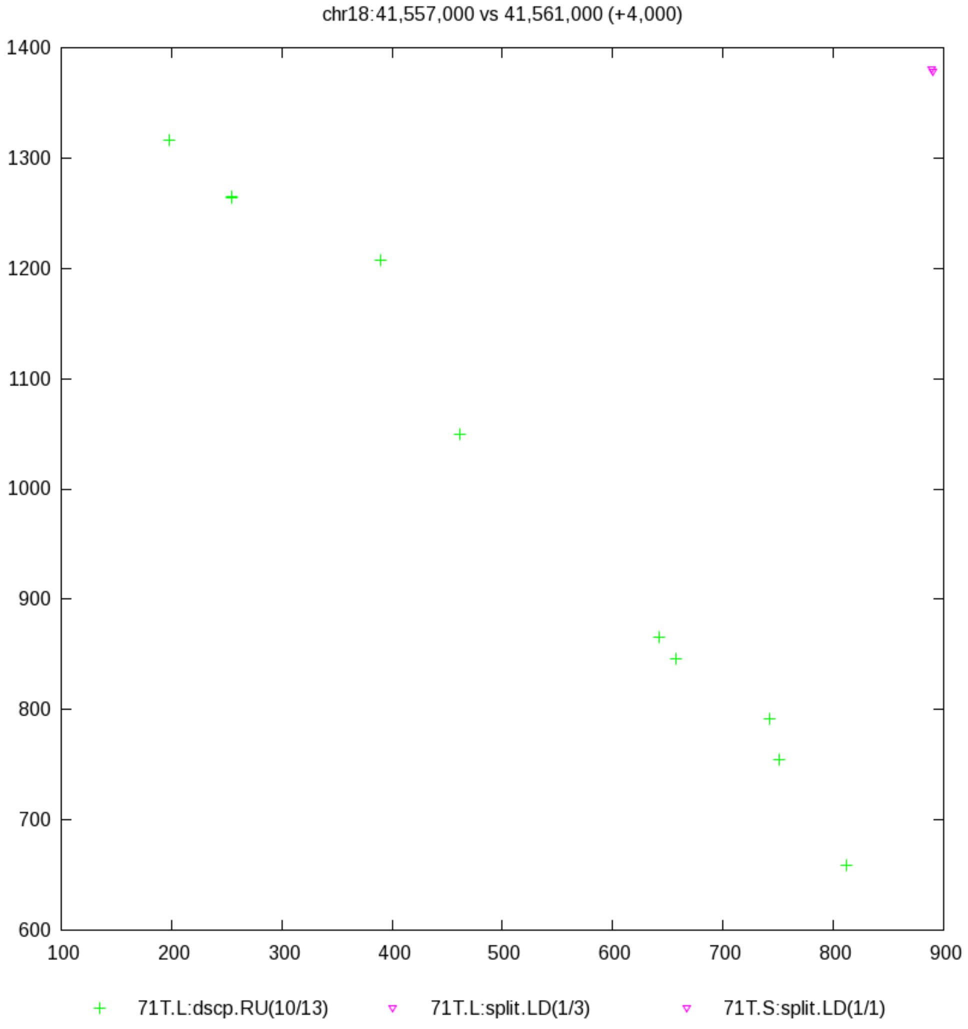

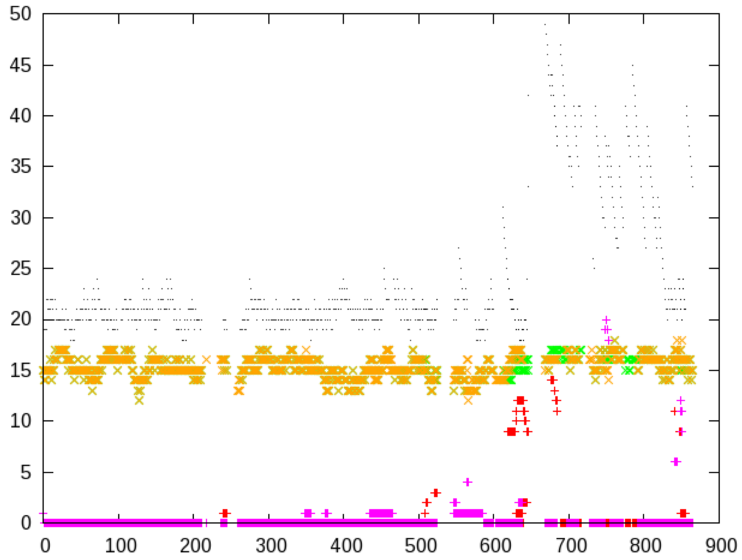

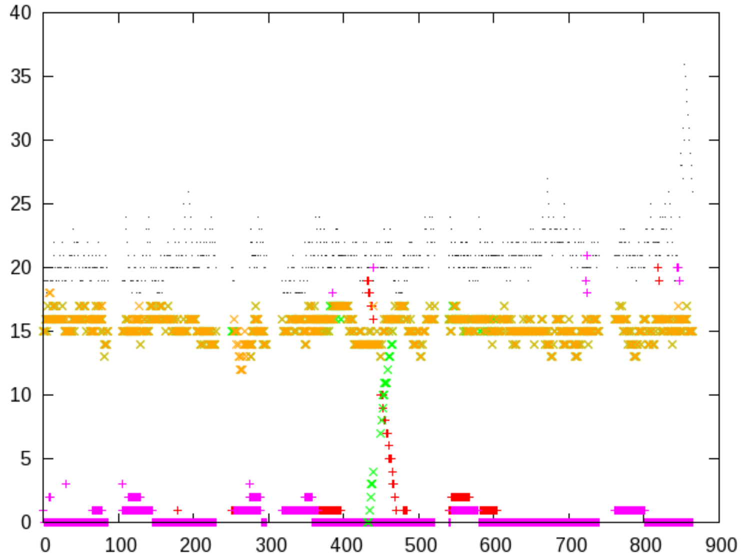

Cluster: 0006

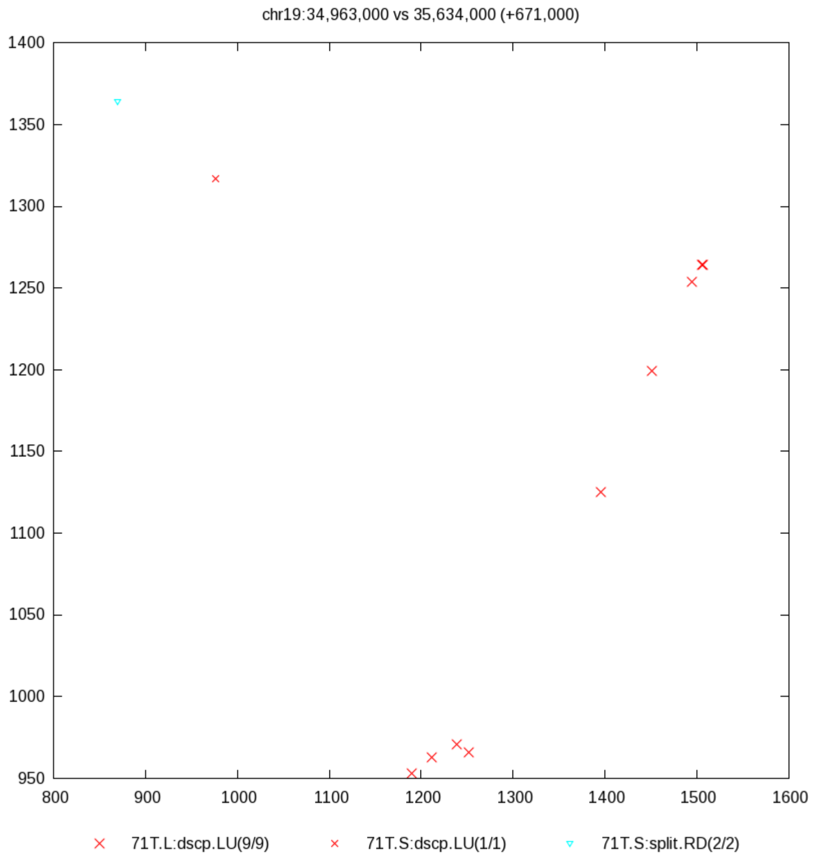

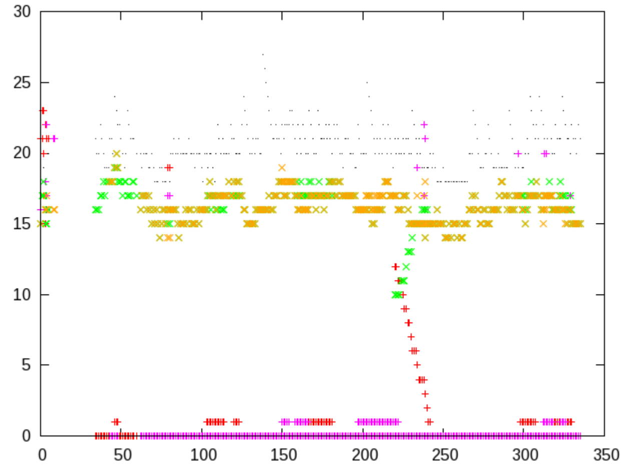

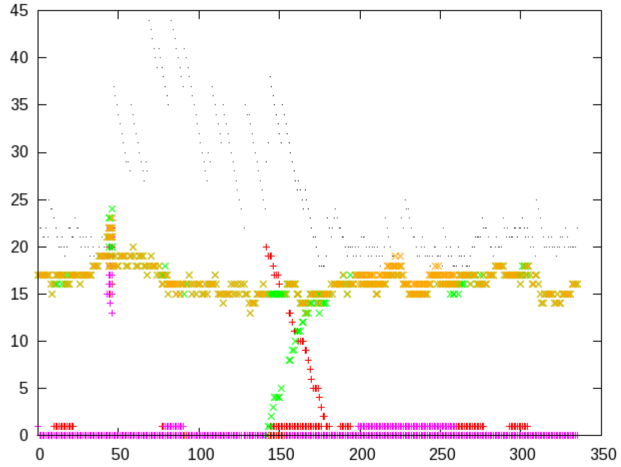

Cluster: 0007

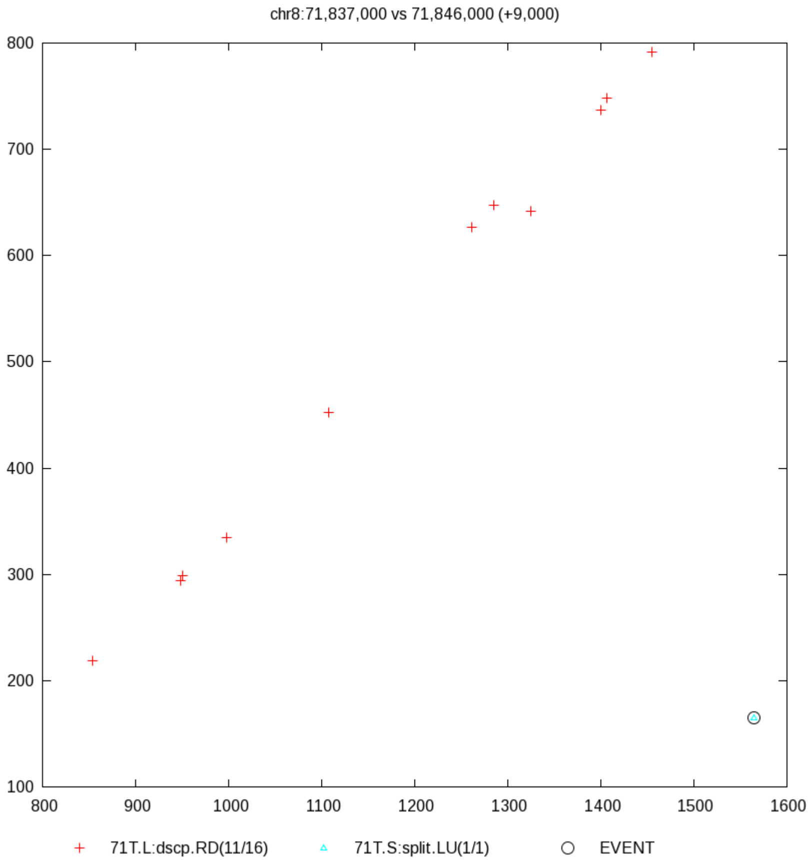

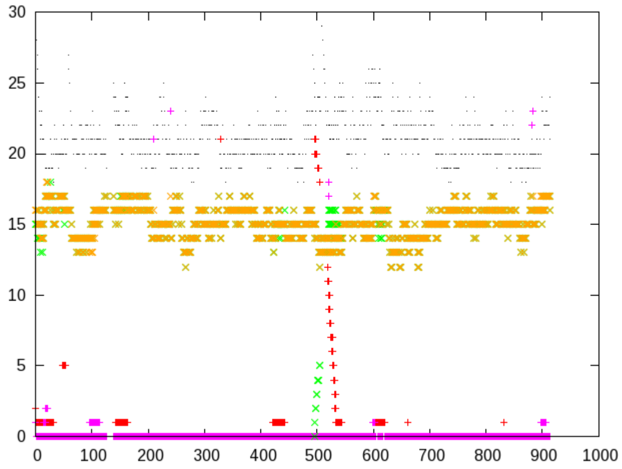

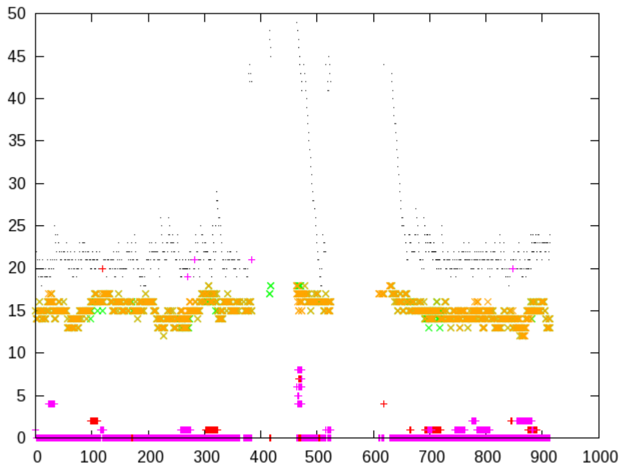

Cluster: 0008

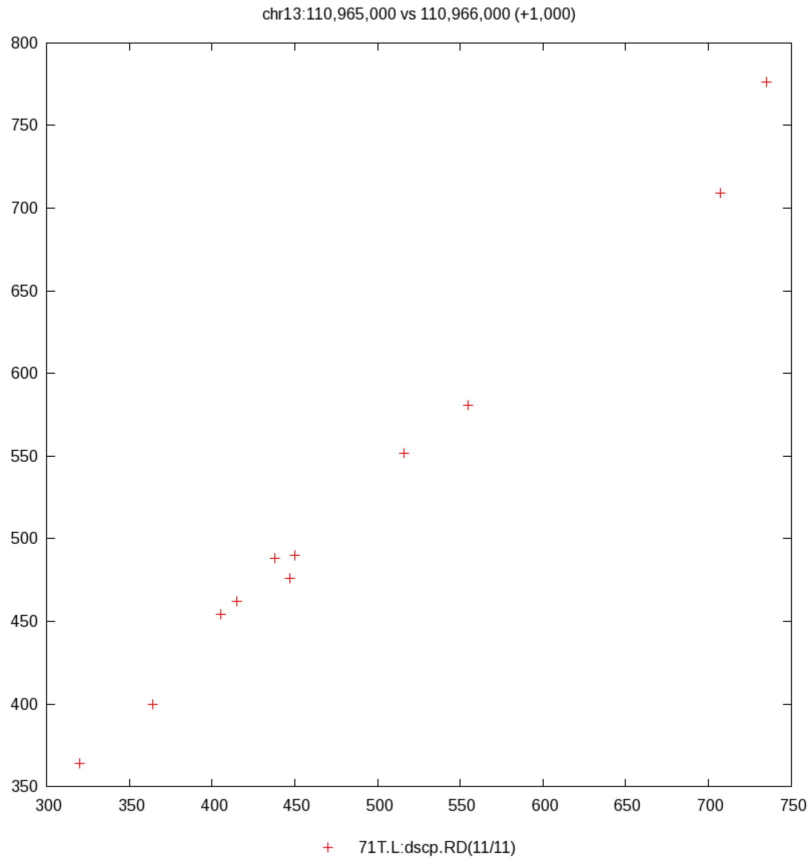

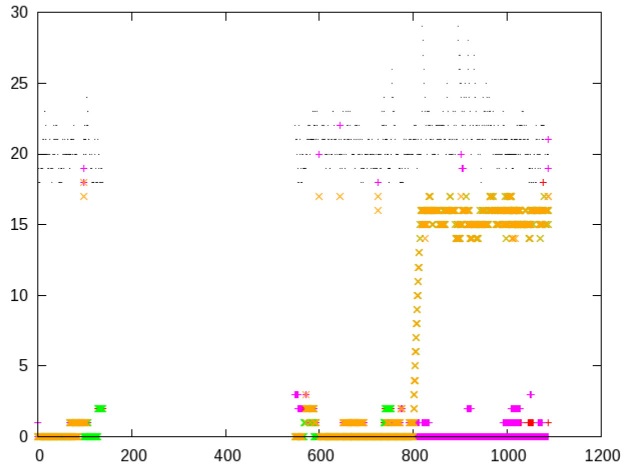

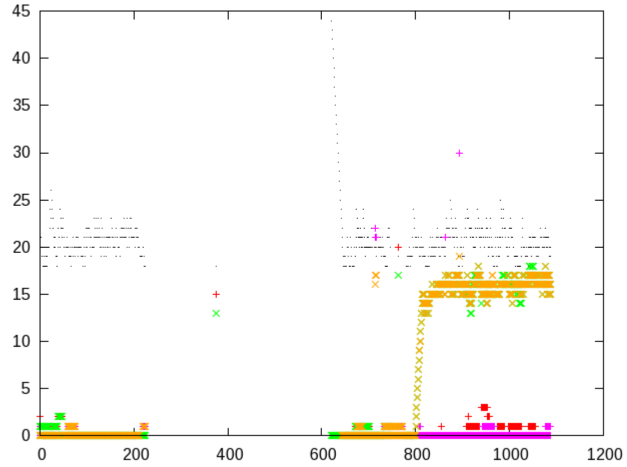

Cluster: 0009

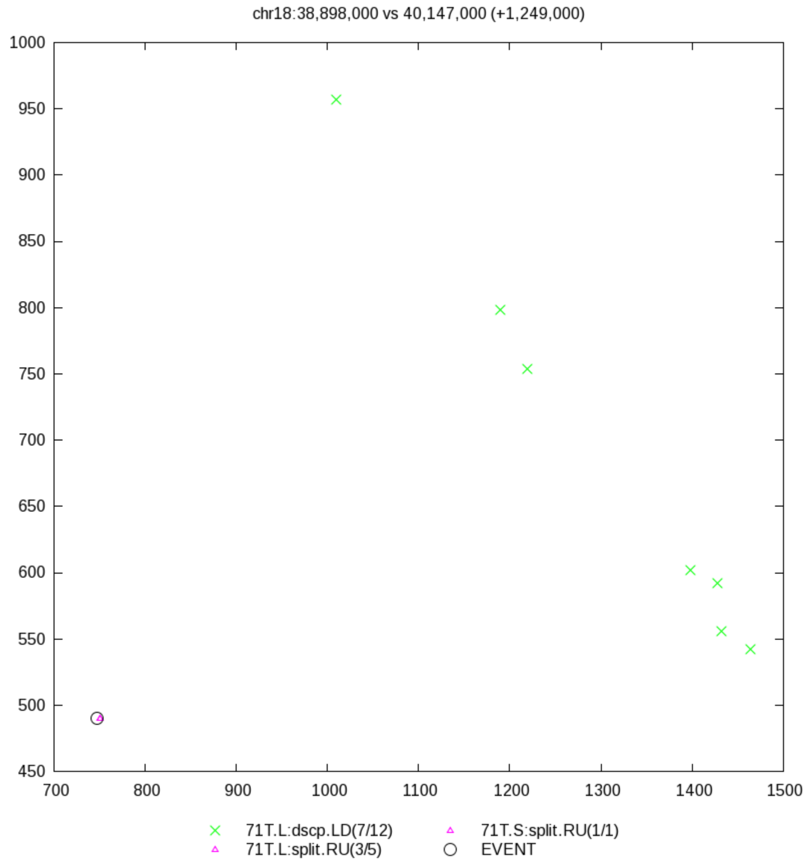

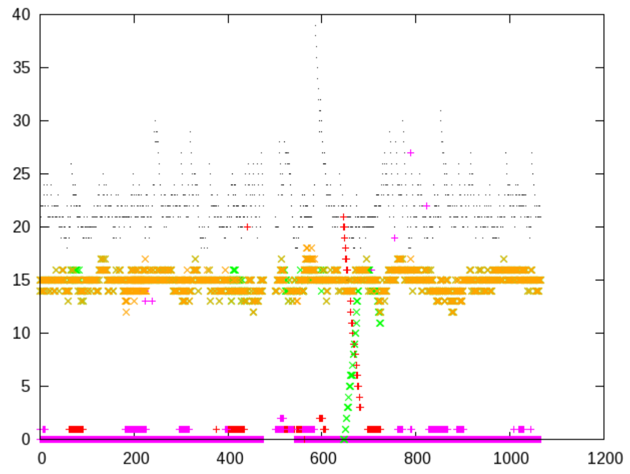

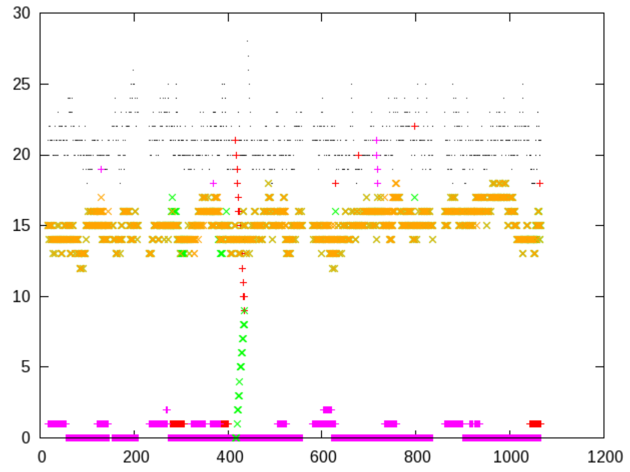

Cluster: 0010

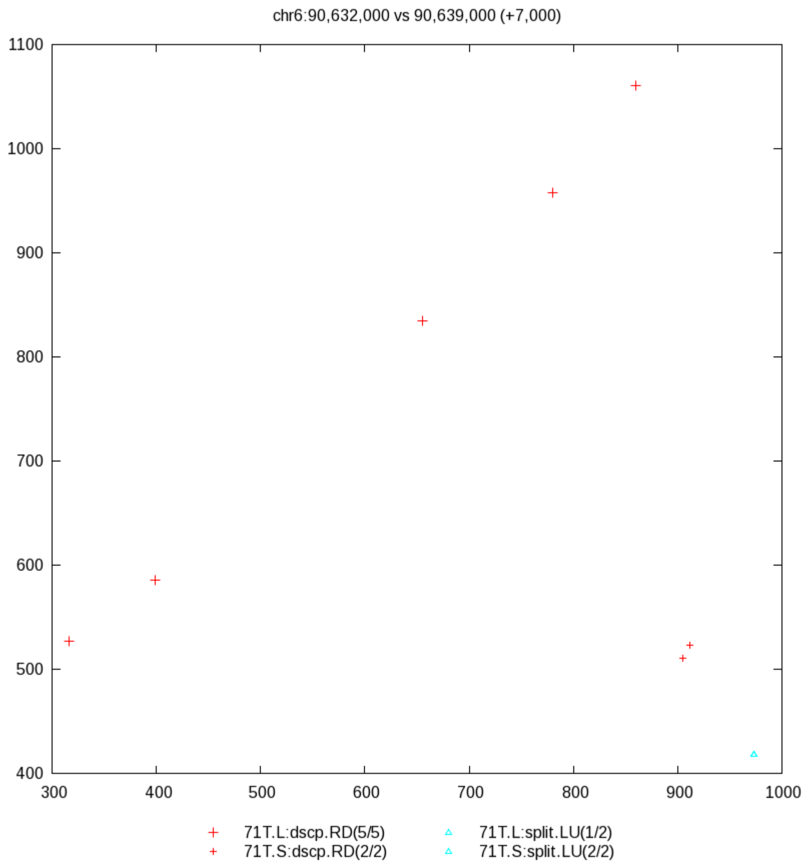

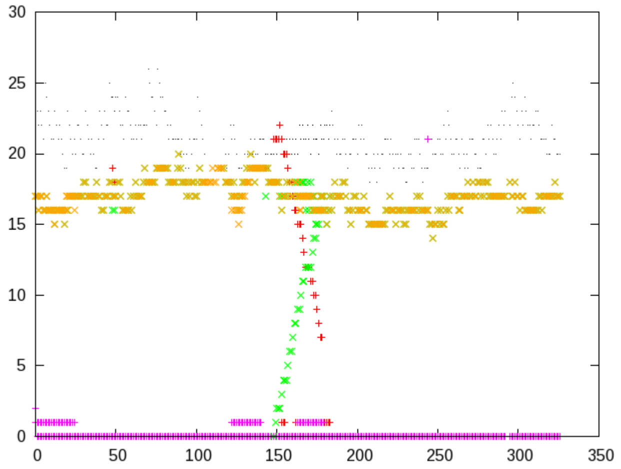

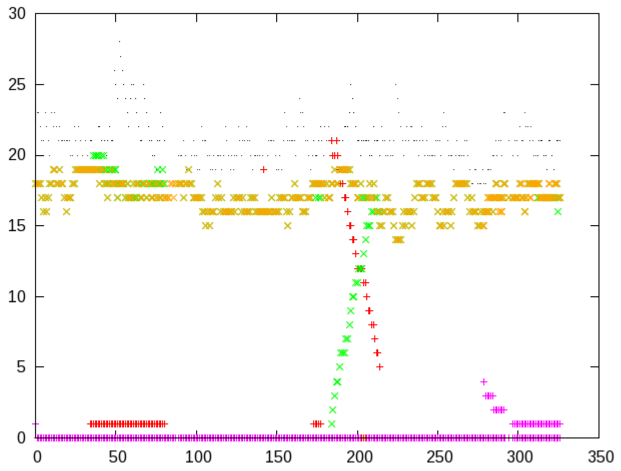

Supplement: Additional file 3 — Evidential materials (2) in the analysis of Patient ID 71. For the same patient, evidential materials for detected somatic intra-chromosomal breakpoints are given. [file 1471-2105-16-S18-S5-S3.pdf]
